# A Neuronal Acetylcholine Receptor Regulates the Balance of Muscle Excitation and Inhibition in *Caenorhabditis elegans*

**DOI:** 10.1371/journal.pbio.1000265

**Published:** 2009-12-22

**Authors:** Maelle Jospin, Yingchuan B. Qi, Tamara M. Stawicki, Thomas Boulin, Kim R. Schuske, H. Robert Horvitz, Jean-Louis Bessereau, Erik M. Jorgensen, Yishi Jin

**Affiliations:** 1 Department of Biology, University of Utah, Salt Lake City, Utah, United States of America; 2 Physiologie Intégrative Cellulaire et Moléculaire, UMR CNRS 5123, Université Lyon 1, Villeurbanne, France; 3 Howard Hughes Medical Institute, University of California San Diego, San Diego, California, United States of America; 4 Division of Biological Science, Section of Neurobiology, University of California San Diego, San Diego, California, United States of America; 5 Ecole Normale Supérieure, Biology Department; INSERM U789, Biologie Cellulaire de la Synapse, Paris, France; 6 Howard Hughes Medical Institute, Massachusetts Institute of Technology, Cambridge, Massachusetts, United States of America; 7 Department of Biology, McGovern Institute For Brain Research, Massachusetts Institute of Technology, Cambridge, Massachusetts, United States of America; 8 Howard Hughes Medical Institute, University of Utah, Salt Lake City, Utah, United States of America; Baylor College of Medicine, United States of America

## Abstract

In the nematode *Caenorhabditis elegans*, cholinergic motor neurons stimulate muscle contraction as well as activate GABAergic motor neurons that inhibit contraction of the contralateral muscles. Here, we describe the composition of an ionotropic acetylcholine receptor that is required to maintain excitation of the cholinergic motor neurons. We identified a gain-of-function mutation that leads to spontaneous muscle convulsions. The mutation is in the pore domain of the ACR-2 acetylcholine receptor subunit and is identical to a hyperactivating mutation in the muscle receptor of patients with myasthenia gravis. Screens for suppressors of the convulsion phenotype led to the identification of other receptor subunits. Cell-specific rescue experiments indicate that these subunits function in the cholinergic motor neurons. Expression of these subunits in *Xenopus* oocytes demonstrates that the functional receptor is comprised of three α-subunits, UNC-38, UNC-63 and ACR-12, and two non–α-subunits, ACR-2 and ACR-3. Although this receptor exhibits a partially overlapping subunit composition with the *C. elegans* muscle acetylcholine receptor, it shows distinct pharmacology. Recordings from intact animals demonstrate that loss-of-function mutations in *acr-2* reduce the excitability of the cholinergic motor neurons. By contrast, the *acr-2(gf)* mutation leads to a hyperactivation of cholinergic motor neurons and an inactivation of downstream GABAergic motor neurons in a calcium dependent manner. Presumably, this imbalance between excitatory and inhibitory input into muscles leads to convulsions. These data indicate that the ACR-2 receptor is important for the coordinated excitation and inhibition of body muscles underlying sinusoidal movement.

## Abstract 2

The role of a heterotrimeric neuronal acetylcholine receptor in regulating a *Caenorhabditis elegans* locomotion circuit are revealed down to the level of identifying all five subunits involved.

## Author Summary

Neuronal acetylcholine receptors modulate a wide range of activities in vertebrates and invertebrates. The activity and sensitivity of these receptors to particular pharmacological agents is determined by the subunit composition of the receptors. A rich diversity of acetylcholine receptors are expressed in the nervous system of the nematode *C. elegans*, and like their mammalian counterparts, their subunit compositions are not understood. Here, we identify an activating mutation in a neuronal acetylcholine receptor subunit that causes convulsive body muscle contractions. By isolating suppressors of the convulsive phenotype, we are able to identify the genes required for the assembly and function of this acetylcholine receptor. Reconstitution studies in oocytes demonstrate that this acetylcholine receptor is composed of five different subunits. The contraction and relaxation of body muscles are coordinated by the neurotransmitters acetylcholine and GABA, respectively. In vivo recordings reveal that loss of this ion channel leads to a decrease in the activation state of the cholinergic motor neurons. By contrast, hyperactivation of the ion channel leads indirectly to the silencing of GABAergic motor neurons. The resulting imbalance in the locomotory circuit causes convulsions of the body muscle. This imbalance in excitation and inhibition of the locomotion circuit mimics the neurological features observed in epilepsy.

## Introduction

Acetylcholine activates ligand-gated ion channels in muscles and is a major neurotransmitter in the brain, modulating a variety of cognitive and addictive behaviors [1]. Ionotropic acetylcholine channels result from the assembly of five individual subunits. Each subunit has four membrane-spanning domains, with the second transmembrane (TM2) domain lining the pore of the channel. Subunits assemble to form a pentameric channel; some subunits, such as α7, can form homopentameric channels [2], but most receptors are heteromeric [3]. For example, the well-studied muscle receptor in mammals contains two ligand-binding α subunits and three non–α-subunits [4],[5]. By contrast, the composition and expression pattern of most neuronal acetylcholine receptors have not been well defined. The subunit composition of a channel and the identity of the pore-lining residues are crucial for ion selectivity, gating, desensitization, ligand affinity, and pharmacology. Because of the diversity of acetylcholine receptor subunits and promiscuous assembly under nonnative conditions, it remains a major challenge to define the in vivo compositions and, consequently, the cell-specific functions of acetylcholine-gated channels.

The genome of the nematode *C. elegans* encodes 29 acetylcholine receptor subunits [6],[7]. The most well-studied receptor is the levamisole-sensitive receptor expressed in the body muscle. The levamisole-sensitive receptor is composed of three α-subunits, UNC-38, UNC-63, and LEV-8, and two non–α-subunits, UNC-29 and LEV-1 [7]–[13]. This receptor functions as the main excitatory postsynaptic receptor at neuromuscular junctions. Genome-wide transgene expression studies indicate that a large number of acetylcholine receptor subunits are expressed in neurons [14]. However, candidate null mutations for many acetylcholine receptor subunits cause few discernable defects. The roles and compositions of most neuronal acetylcholine receptors remain unknown, and reconstitution experiments have not been performed.

In this study, we identify all five subunits of a neuronal acetylcholine receptor and characterize its function in a neural circuit controlling *C. elegans* locomotion. Our approach relied on a mutant strain exhibiting severe convulsions due to overstimulation of the muscles. Molecular characterization demonstrated that this mutant strain possessed an activating mutation in an acetylcholine receptor subunit, ACR-2. We identified the other components of the ACR-2 receptor by screening for second-site mutations that ameliorated the convulsive phenotype. Combined with cell-type expression studies and receptor reconstitution in *Xenopus* oocytes, these data led to a complete description of the subunit composition of a neuronal acetylcholine receptor. We further demonstrated that the ACR-2 receptor functions to maintain excitability of the cholinergic neurons, by recording synaptic activity in the null and activated mutants. The hyperactivating ACR-2 mutation leads to enhanced neurotransmitter release from the cholinergic motor neurons, and intriguingly, an inactivation of the GABAergic motor neurons that receive inputs from the cholinergic motor neurons. The imbalance in the excitation and inhibition within the motor circuit disrupts coordinated body muscle contraction.

## Results

### An Activated Acetylcholine Receptor Subunit

*C. elegans* crawls by generating a sinusoidal wave that is propagated from the head to the tail. These contractions are generated by acetylcholine released from ventral cord motor neurons. The cholinergic motor neurons form dyadic synapses, simultaneously innervating the musculature and GABAergic motor neurons [15]. The GABAergic motor neurons form neuromuscular junctions on the opposite side of the animal and thereby relax muscles on the opposite side of the body. In the absence of GABA neurotransmission *C. elegans* hypercontract or “shrink” when stimulated to move by gentle touch [16]. We isolated the mutation *n2420* in a screen for mutants that shrink in response to gentle touch. However, *n2420* worms have an additional phenotype not expressed by classic shrinker mutants: they shrink spontaneously, referred to here as a “convulsion” (Video S1). Thus, the mutant combines the classic phenotype of a loss of GABA function with spontaneous activation of muscle contraction. The convulsion phenotype is recessive, although *n2420* is a semidominant gain-of-function mutation (described below).

**Figure 1:**
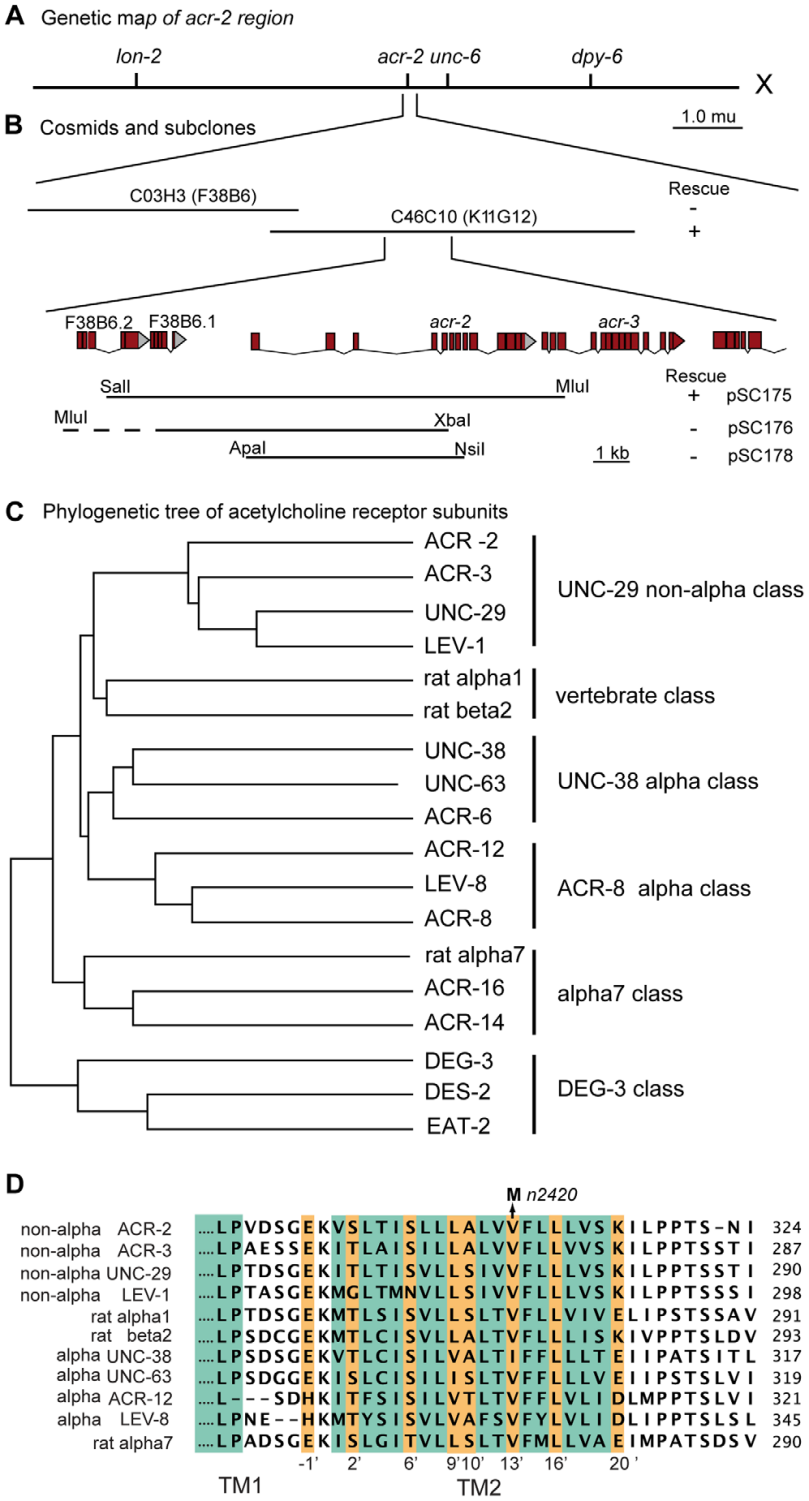
*acr-2* locus. (A) Genetic position of *acr-2* on the left arm of the X chromosome. (B) Representative cosmids and subclones used in germline transformation rescue of *acr-2(n2420gf)*. Rescue of spontaneous convulsion is indicated by a plus sign (+); and no rescue by a minus sign (−). Canonical cosmids F38B6 and K11G12 are shown in parentheses. (C) ACR-2 is a member of the UNC-29 class non–α-subunits of the acetylcholine receptor family [14]. (D) The *n2420* mutation causes a valine-to-methionine change in the 13′ position of the pore-forming second transmembrane domain (TM2, residues are colored in teal). The amino acid at the 13′ position is oriented toward the pore; orange mark residues lining the pore [20].

We mapped *n2420* between *lon-2* and *unc-6* on chromosome X (Figure 1A). Microinjection of the cosmid C46C10 rescued both the spontaneous convulsions and uncoordinated behavior of *acr-2(n2420)* animals. The rescuing activity was further narrowed to a 10-kb DNA fragment containing the *acr-2* gene (Figure 1B). The *acr-2* gene encodes a 580–amino acid subunit of an ionotropic acetylcholine receptor [17]. The predicted genomic organization was confirmed by the sequences of four cDNAs that correspond to the *acr-2* locus (see Materials and Methods). *acr-2* is the upstream gene in an operon, with the closely related gene *acr-3* immediately downstream (Figure 1B) [18]. ACR-2 and ACR-3 are non–α-subunits in the heteromeric receptor clade. They are closely related to the UNC-29 and LEV-1 subunits of the levamisole-sensitive receptor and more distantly to the vertebrate heteromeric receptor subunits (Figure 1C).

**Figure 2:**
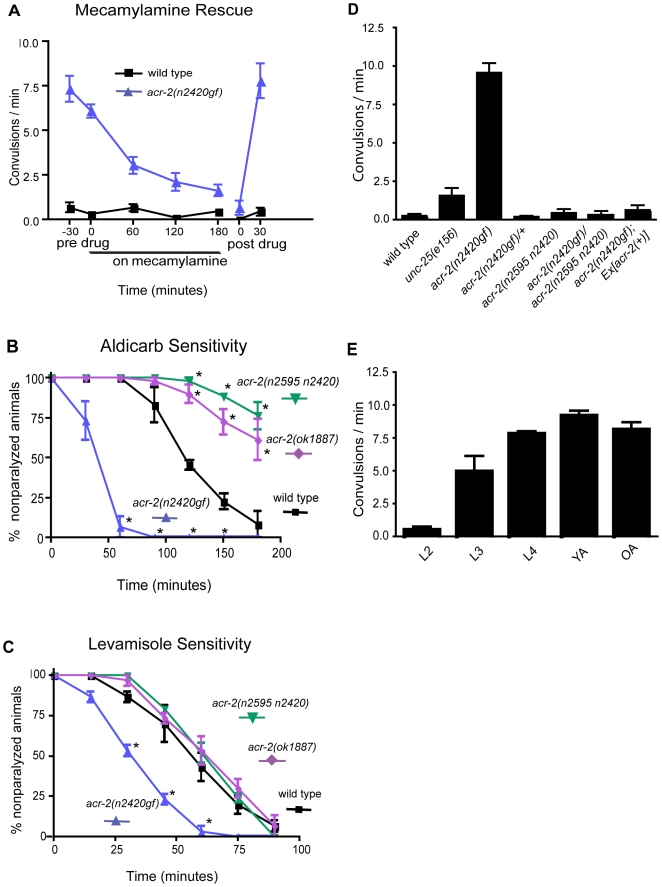
Mutant phenotypes and pharmacological analysis of *acr-2(n2420gf)* mutants. (A) Suppression of the convulsions of *acr-2(n2420gf)* mutants by exogenous mecamylamine (100 µM). The effects of mecamylamine are reversible. (B) *acr-2(n2420gf)* mutants are hypersensitive, and *acr-2(lf)* mutants are moderately resistant to aldicarb. The graph shows the time course of the response of adult hermaphrodites to 0.5 mM aldicarb, from three trials with ten animals per genotype per trial. *p<0.001 between wild type and mutants (two-way ANOVA). (C) *acr-2(n2420gf)* mutants are hypersensitive to levamisole. The graph shows the time course of the response of 1-d-old hermaphrodites to 1 mM levamisole, from three trials with ten animals per genotype per trial. *p<0.001 between wild type and *acr-2(n2420gf)* (two-way ANOVA). (D) Quantification of convulsion rates of 1-d-old hermaphrodites of various genotypes. (E) Developmental onset of the convulsion phenotype of *acr-2(n2420gf)* animals occurs during the L3 stage. OA, 2-d-old adult; YA, 1-d-old adult.

The molecular lesion of *acr-2(n2420)* is consistent with its being a gain-of-function mutation in the ion channel. The mutation in *n2420* results in a valine 309 to methionine substitution, which is at the 13′ position in the pore-forming TM2 domain of the ACR-2 subunit (TM2 numbering scheme as in [19]) (Figure 1D). The 13′ position is thought to line the pore and contribute to ion selectivity [20]. A similar mutation at the 13′ position leads to neurodegeneration in the neuronal acetylcholine receptor DEG-3 [21]. A corresponding mutation in the β-subunit of the muscle acetylcholine receptor is found in human patients with myasthenia gravis [22]. This substitution generates a receptor with increased sensitivity to acetylcholine, prolonged open times, and spontaneous activity in the absence of acetylcholine [22]. The pharmacological responses of the *n2420* strain are consistent with an activation mutation in an acetylcholine receptor. The convulsions were reversibly suppressed by mecamylamine (Figure 2A, Video S2), a noncompetitive open-channel blocker [23]. The mutant animals were hypersensitive to aldicarb (Figure 2B), an acetylcholinesterase inhibitor that prolongs endogenous acetylcholine stability in the synaptic cleft [7]. *acr-2(n2420)* animals were also hypersensitive to the acetylcholine agonist levamisole (Figure 2C), which activates a class of acetylcholine receptors expressed on nematode muscles. The hypersensitivity to both aldicarb and levamisole has been observed in several mutants that exhibit an increased level of acetylcholine release and a reduced level of GABA release onto the muscle [24].

The *n2420* mutation genetically behaves as a weak semidominant mutation. Heterozygous *n2420* animals (*n2420/+*) are slightly uncoordinated when moving backward. The dominant phenotype is not caused by haploinsufficiency of this interval, since hemizygous animals carrying a deficiency of the region in *trans* to a wild-type chromosome (+/*Df*) move normally (see Materials and Methods). Nevertheless, the convulsion phenotype of *n2420* mutants is recessive (Figure 2D). Homozygotes of *n2420* contract spontaneously at a frequency of 9±1 convulsions per minute, whereas heterozygotes of *n2420/+* do not exhibit convulsions. In fact, animals that are heterozygous for *acr-2(n2420)* and a deficiency of the region (*n2420*/*Df*) (see Materials and Methods) or a null mutation (Figure 2D and described below) also lack spontaneous convulsions, indicating that the mutation causes altered gene function [25]. The convulsion behavior is not observed in young larvae and is first visible at the L3 stage (Figure 2E).

### ACR-2 Regulates Excitation of Cholinergic Motor Neurons

**Figure 3:**
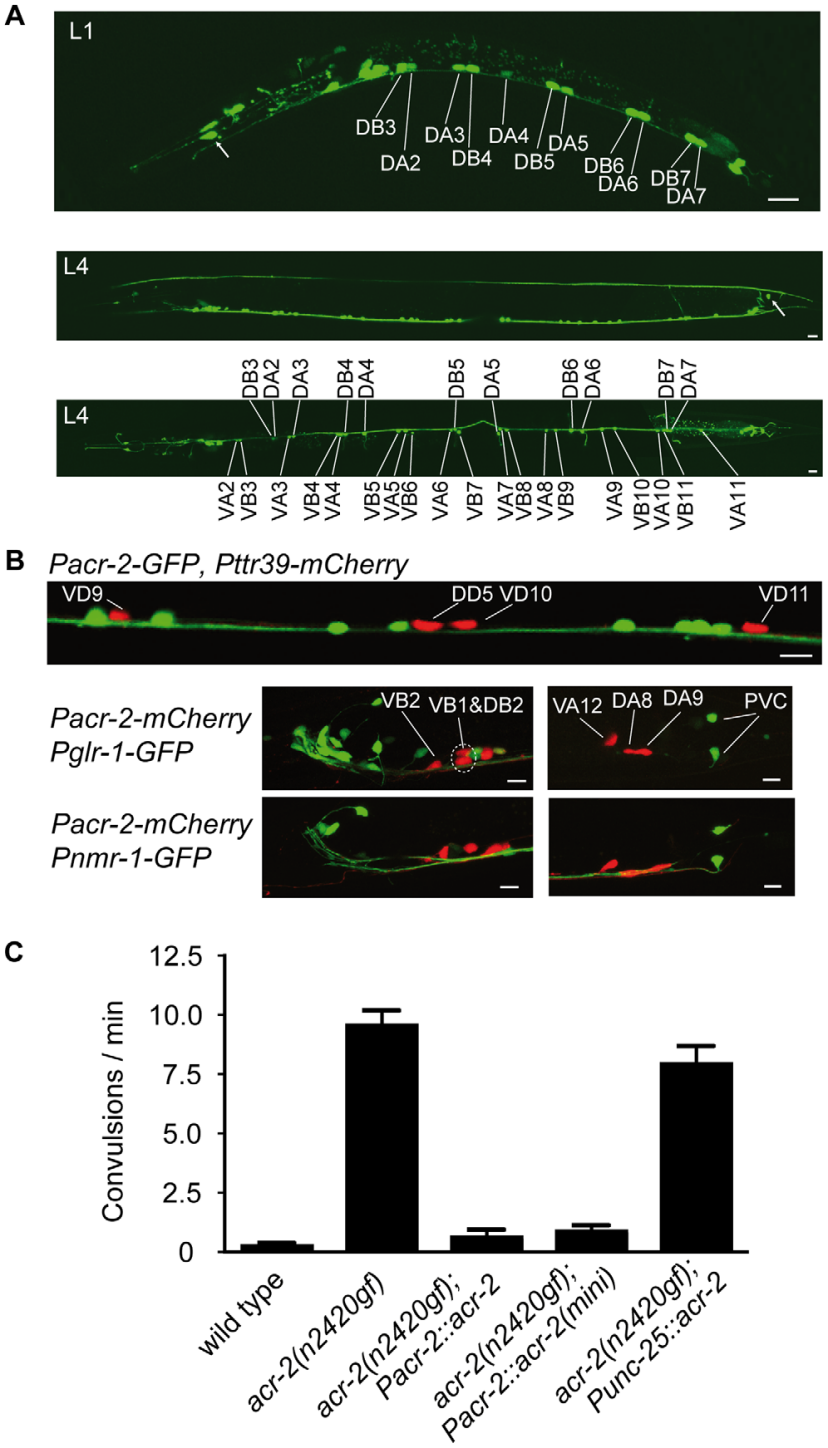
*acr-2* is expressed and functions in the cholinergic ventral cord motor neurons. (A) Expression pattern from a 3.5-kb *acr-2* promoter-driven *Pacr-2-GFP* transgene (*juIs14*) in an L1 (upper image) and an L4 larva. Middle image is a side view, and the bottom image is a ventral view of an L4 larva. The number and positions of the cells and the sides of the commissures indicate that they are embryonically born DA and DB and postembryonically born VA and VB neurons. Expression is also seen in DVC in the tail (arrow, middle panel). Scale bars indicate 10 µm. (B). The top image shows nonoverlapping expression of *Pacr-2-GFP* (*juIs14*) (green) and *Pttr-39-mcherry* (*juIs223*) (red) marking the DD and VD GABAergic neurons. The bottom images show nonoverlapping expression of the 1.8-kb promoter-driven *Pacr-2-mcherry* transgene (*juEx2045*) (red) and those of *Pglr-1-GFP* (*nuIs1*) (green) or *Pnmr-1-GFP* (*akIs3*) (green), which label the command interneurons in the head (left panels) and tail (right panels) ganglia. Transcriptional activity of the *acr-2* 1.8-kb promoter is not seen in any head neurons. Scale bars indicate 10 µm. (C) Quantification of cell-type–specific transgenic rescue of the convulsion defects in *acr-2(n2420gf)* animals. *Pacr-2::acr-2* contains the full-length genomic coding sequence driven by the 3.5-kb-long promoter. P*acr-2::acr-2(mini)* contains cDNA that replaced genomic DNA from exon 2, driven by the 1.8-kb promoter. *Punc-25::acr-2* contains the full-length genomic coding sequences driven by the 1.4-kb *unc-25* promoter. *n* = 10, 10, 5, 10, and 8 for N2, *acr-2(n2420gf)*, *acr-2(n2420gf);Pacr-2-acr-2(oxEx707)*, *acr-2(n2420gf); Pacr-2-acr-2(mini) (juEx2336)*, and *acr-2(n2420gf);Punc-25-acr-2(juEx32)*, respectively.

To determine the site of action of *acr-2*, we constructed transcriptional reporter genes by placing the fluorescent proteins GFP or mCherry under the control of a 3.5-kb or a 1.8-kb *acr-2* promoter region (see Materials and Methods). Both promoters drove expression predominantly in the neurons of the ventral cord from L1 larvae to adults (Figure 3A). Based on their birth times, numbers, positions, and axon morphologies, these neurons were determined to be cholinergic motor neurons of the VA, VB, DA, and DB classes, and not the AS and VC classes [26]. GFP expression driven by the longer *acr-2* promoter was frequently seen in the PVQ and DVC neurons in the tail and was infrequently observed in a few head neurons (Figure 3A). Coexpression with reporter genes for GABAergic neurons and interneurons confirmed that the *acr-2* transcriptional reporters were not expressed in the ventral cord GABAergic motor neurons or in the interneurons expressing *glr-1* or *nmr-1* (Figure 3B). The *acr-2(n2420gf)* convulsion defect was not rescued when we expressed the ACR-2 protein in the GABAergic neurons (Figure 3C). Expression of an *acr-2* mini-gene in which the genomic sequences from exon 2 through 3′ UTR was replaced by *acr-2* cDNA, driven under the 1.8-kb short promoter, could rescue the convulsion defect to a similar degree as the full-length *acr-2* (Figure 3C). These data suggest that ACR-2 functions in the cholinergic ventral cord motor neurons.

**Figure 4:**
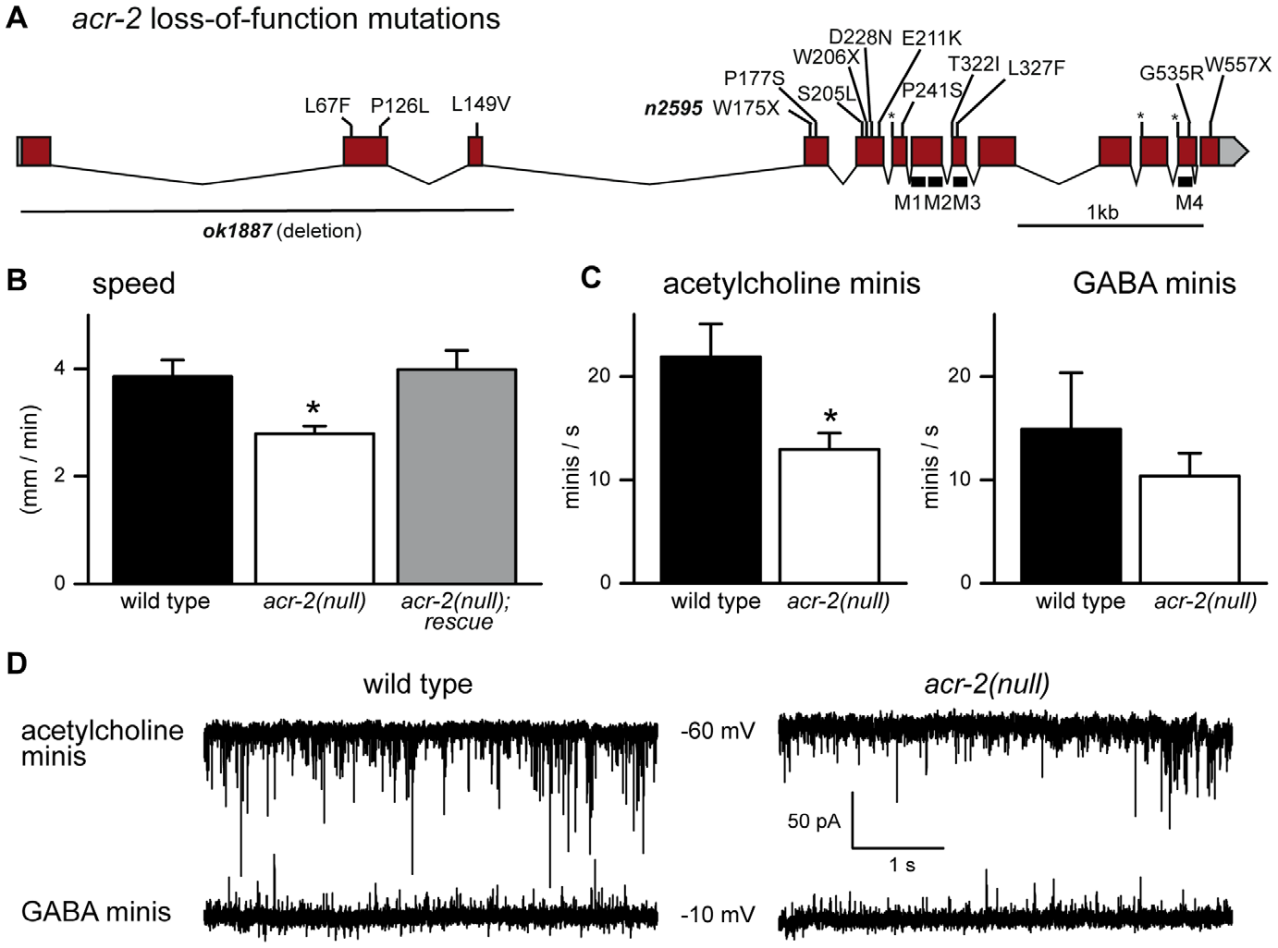
Acetylcholine neurotransmission is reduced in *acr-2* null mutants. (A) *acr-2* gene structure with the intragenic mutations identified from the *acr-2(n2420gf)* suppressor screen. *acr-2(n2595 n2420)* contains a nonsense mutation at the codon for tryptophan 175 in addition to the original gain-of-function mutation and is used as the null mutant in this figure. *ok1887* removes the first three exons and inserts 420 bp of unrelated DNA. Details of the nucleotide and amino acid changes are shown in Table S1 and Figure S1. Boxes indicate exons; lines, introns; M, transmembrane domain; an asterisk (*) indicates mutations at the splice junctions; X indicates stop codon mutations. (B) Average speed of wild-type (3.9 mm/min±0.3 SEM, *n* = 16), *acr-2(n2595 n2420)* (2.8 mm/min±0.1 SEM, *n* = 16), and *acr-2(n2595 n2420)* worms carrying a *Pacr-2::acr-2* construct (4.0 mm/min±0.4 SEM, *n* = 16). An ANOVA test followed by a Dunn's post test was used to analyze the data; *p<0.05. (C) Acetylcholine mini frequencies recorded in 2 mM external CaCl_2_ from the wild type (21.9 events/s±3.2 SEM, *n* = 5) and *acr-2(n2595 n2420)* (12.9 events/s±1.6 SEM, *n* = 9) are significantly different (*p = 0.015). GABA mini frequencies recorded in 2 mM external CaCl_2_ from the wild type (14.9 events/s±5.4 SEM, *n* = 5) and *acr-2(n2595 n2420)* (10.4 events/s±2.2 SEM, *n* = 9) are not significantly different (*p* = 0.38). Data were analyzed using a two-tailed unpaired *t*-test. (D) Representative traces of minis recorded at two holding potentials, −60 and −10 mV, on body muscle cells from wild-type and *acr-2(n2595 n2420)* worms in 2 mM external CaCl_2_.

We next sought to obtain null mutations in *acr-2* by performing a genetic screen for suppressors of *acr-2(n2420gf)* convulsions (see Materials and Methods). We identified 30 suppressors that exhibited dominant suppression of the convulsion (Figure 2D). They were mapped to the X chromosome and were found to be tightly linked to *acr-2(n2420gf)*. Sequencing of the DNA revealed that 26 of these linked suppressors contained mutations in the *acr-2* gene itself (Figure 4A, Table S1). Mutations in the other four linked suppressor strains have not yet been identified in the *acr-2* or in the immediately downstream *acr-3* open reading frames. The identification of *acr-2* intragenic second-site suppressor mutations further supports the conclusion that *acr-2(n2420gf)* results in a hyperactive receptor.

The analysis of the molecular lesions in the *acr-2* intragenic mutations indicates that eight are likely to be strong lossof-function or null mutations, since they introduce stop codons or alter splice junctions that would result in truncated or nonfunctional products. For example, two independent isolates (*n2595* and *n2651*) introduce an opal stop at Trp175 about halfway through the extracellular domain (Figures 4A and S1), likely representing null mutations in *acr-2*. Among the other 16 intragenic revertants with amino acid substitutions, 13 affect amino acid residues in the extracellular domain (Figure 4A). Some of these mutations alter candidate ligand-binding residues (such as *n2603* and *n2581*), consistent with the idea that these intragenic revertants abolish the activity of *acr-2(n2420gf)*. Two intragenic mutations (*n2594* and *n2604*) are located in the M2-M3 linker, a region that affects gating of acetylcholine receptors [27].

The *acr-2* loss-of-function mutants are healthy but exhibit slightly sluggish locomotion. The speed of young adult animals was determined using a worm-tracking system. The average velocity of *acr-2(n2595 n2420)* worms was reduced by 28% compared to that of the wild type (Figure 4B). This defect was not caused by background mutations, since this phenotype could be rescued by a wild-type transgene. We also obtained a deletion allele *acr-2(ok1887)* that removes the 5′ region of the gene (Figure 4A). By movement, *acr-2(ok1887)* was indistinguishable from *acr-2(n2595 n2420)*. Both *acr-2(n2595 n2420)* and *acr-2(ok1887)* mutants were fully sensitive to levamisole, the muscle receptor agonist, but were moderately resistant to aldicarb (Figure 2B and 2C). The overall axon morphology and synapses of the cholinergic motor neurons are grossly normal in *acr-2(lf)* animals (unpublished data). These data suggest an impairment in acetylcholine release in *acr-2* null mutants.

To determine the nature of the synaptic defect in *acr-2* null mutants, we performed patch-clamp recordings of the muscle under voltage-clamp conditions. Acetylcholine release was monitored by quantifying miniature postsynaptic currents (“minis”). Each miniature current is caused by neurotransmitter release from a single, or very few, synaptic vesicles from a motor neuron. Under standard conditions, acetylcholine and GABA currents are isolated pharmacologically [28]; however, we wished to preserve interactions between cholinergic and GABAergic motor neurons, so we used recording conditions in which these inputs into muscles could be distinguished without resorting to the use of drugs. Specifically, intracellular solutions were used in which the equilibrium potential for chloride was −59 mV, and the equilibrium potential for cations was 0 mV (see Materials and Methods). At −60 mV, acetylcholine-induced minis would be inward currents, and GABA-induced minis not distinguishable, whereas at −10 mV, GABA-induced minis would be outward currents, and acetylcholine-induced minis would be very small inward currents. In the presence of 2 mM calcium in the external solution, the frequency of acetylcholine minis was reduced to 60% in *acr-2(n2595 n2420)* mutants and 62% in *acr-2(ok1887)* mutants compared to that of the wild type (Figures 4C, 4D, S3A, and S3B). In addition, GABA mini frequency was reduced to 70% in *acr-2(n2595 n2420)* and 67% in *acr-2(ok1887)* mutants compared to the wild type, although this reduction did not reach significance (Figures 4C, 4D, S3A, and S3B). The amplitude of minis remained unchanged in both mutants (Figures S2 and S3C). These results indicate that in the absence of *acr-2* function, neurotransmission from the cholinergic motor neurons is impaired, leading to a weak defect in locomotion. The cholinergic motor neurons form synapses onto the GABAergic motor neurons [15]. The mild effect on GABA neurotransmission might be an indirect effect of reduced excitation from the cholinergic motor neurons.

### *acr-2* Gain-of-Function Mutation Increases Acetylcholine and Decreases GABA Transmission

**Figure 5:**
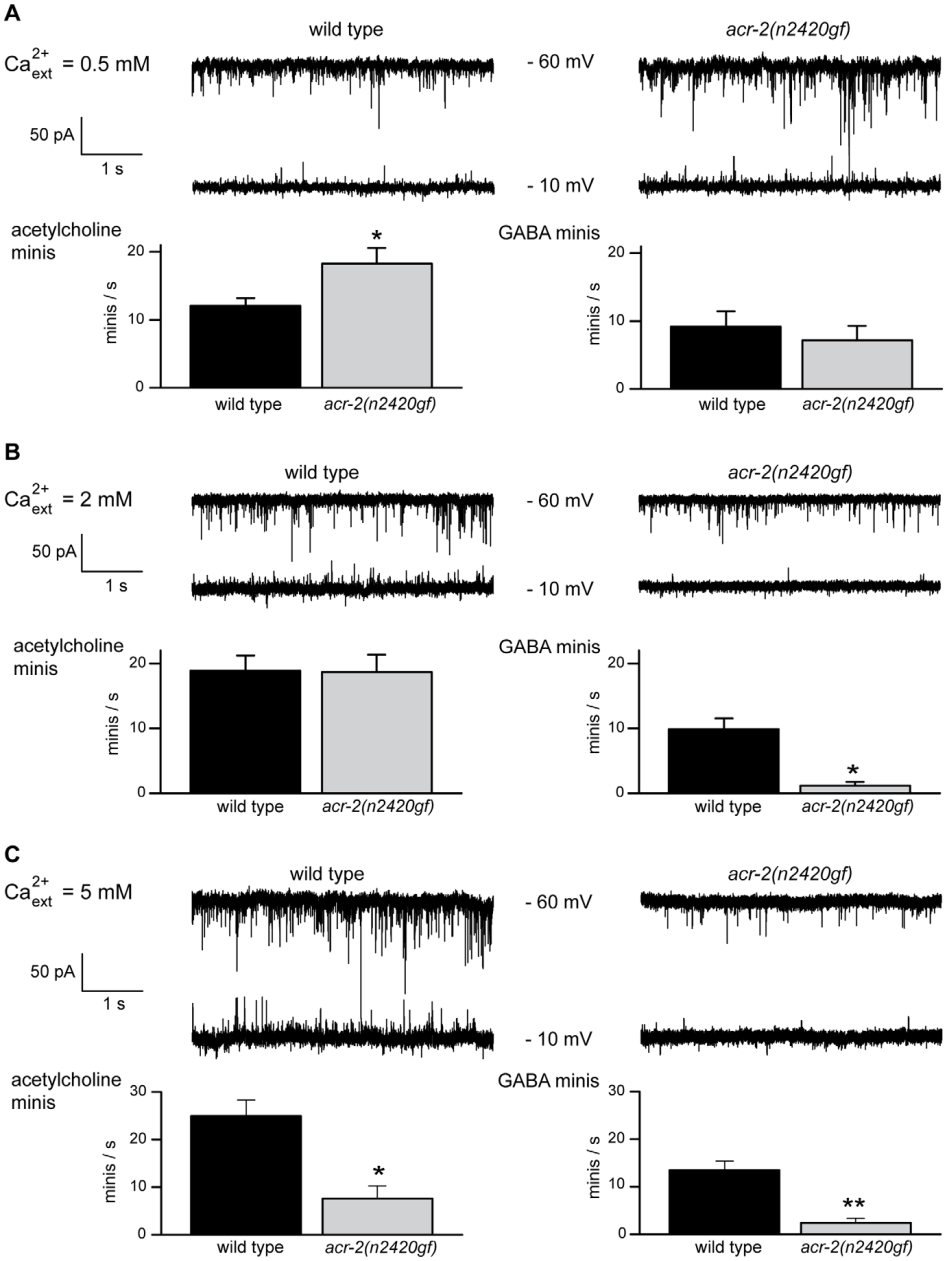
*acr-2(n2420gf)* mutants exhibit reduced GABA neurotransmission. (A) Representative traces (upper panel) and frequencies (lower panel) of endogenous postsynaptic currents recorded at two holding potentials, −60 and −10 mV, from wild-type and *acr-2(n2420gf)* worms in 0.5 mM external CaCl_2_. The frequency of miniature postsynaptic currents from cholinergic neurons is increased in the gain-of-function mutant (wild type: 12.1 events/s±1.1 SEM, *n* = 8; *acr-2(n2420gf)*: 18.3 events/s±2.2 SEM, *n* = 9; *p = 0.0307). The frequency of miniature currents induced by GABAergic motor neurons was only slightly reduced in the gain-of-function mutant (wild type: 9.2 events/s±2.3 SEM, *n* = 8; *acr-2(n2420gf)*: 7.2 events/s±2.1 SEM, *n* = 9; *p* = 0.5342). Data were analyzed using a two-tailed unpaired *t*-test. (B) Representative traces (upper panel) and frequencies (lower panel) of endogenous postsynaptic currents recorded at two holding potentials, −60 and −10 mV, from wild-type and *acr-2(n2420gf)* worms in 2 mM external CaCl_2_. Acetylcholine currents frequencies recorded from the wild type (18.9 events/s±2.3 SEM, *n* = 10) and *acr-2(n2420gf)* (18.7 events/s±2.6 SEM, *n* = 14) are not significantly different (*p* = 0.9555). GABA currents recorded from the wild type (9.9 events/s±1.7 SEM, *n* = 10) and *acr-2(n2420gf)* (1.2 events/s±0.6 SEM, *n* = 14) are significantly different (*p = 0.0007). Data were analyzed using a two-tailed unpaired *t*-test with Welch's correction. (C) Representative traces (upper panel) and frequencies (lower panel) of endogenous postsynaptic currents recorded at two holding potentials, −60 and −10 mV, from wild-type and *acr-2(n2420gf)* worms in 5 mM external CaCl_2_. Acetylcholine currents frequencies recorded from the wild type (24.9 events/s±3.4 SEM, *n* = 8) and *acr-2(n2420gf)* (7.6 events/s±2.7 SEM, *n* = 7) are significantly different (*p = 0.0017). GABA currents recorded from the wild type (13.4 events/s±2.0 SEM, *n* = 8) and *acr-2(n2420gf)* (2.3 events/s±1.0 SEM, *n* = 7) are significantly different (**p = 0.0004). Data were analyzed using a two-tailed unpaired *t*-test.

Since loss of *acr-2* function causes a decrease in cholinergic motor neuron activity, then it is likely that the gain-of-function mutation hyperactivates cholinergic neurons. The hypersensitivity of *acr-2(n2420gf)* animals to aldicarb (Figure 2B) is consistent with this idea. Our recordings of endogenous mini currents in the dissected preparation supported this prediction, but we also found that hyperactivity of the cholinergic neurons was highly sensitive to calcium. We recorded the frequency of acetylcholine-induced mini currents at three concentrations of calcium: 0.5 mM, 2 mM, and 5 mM. At 0.5 mM calcium, the acetylcholine mini frequency in *acr-2(n2420gf)* animals was 150% compared to the wild type (Figure 5A), demonstrating that the altered pore domain of this mutant receptor caused an increase in activity in cholinergic neurons. In 2 mM extracellular calcium, the frequency of acetylcholine minis in *acr-2(n2420gf)* was not increased but was similar to the wild type (Figure 5B). At 5 mM calcium, mini frequency in the *acr-2(n2420gf)* cholinergic neurons was reduced to 30% compared to the wild type (Figure 5C). Thus, in *acr-2(n2420gf)* animals, the cholinergic neurons are more active than those in the wild type at low levels of calcium, but these motor neurons are inhibited at very high levels of calcium in the mutant. No discernable abnormalities in morphology and synapses of the cholinergic motor neurons were observed in *acr-2(n2420gf)* animals (Figure S4). Hyperactive acetylcholine neurotransmission is a likely cause of the spontaneous muscle contractions observed in the gain-of-function *acr-2* mutant.

In addition to spontaneous muscle contraction, *acr-2(n2420gf)* mutants behave as though they are impaired for GABA transmission, that is, they shrink when touched. The shrinking behavior could be the result of GABAergic neuron developmental defects [29]. Neurogenesis and axon morphology were normal based on GFP expression in the GABAergic neurons (unpublished data). Synaptic development was assayed by quantifying synaptic varicosities in the GABAergic motor neurons. Synaptic vesicle clusters were marked by expressing GFP-tagged synaptobrevin in the GABAergic neurons and fluorescent puncta along the ventral cord were counted. The number of fluorescent clusters was similar in *acr-2(n2420gf)* animals and the wild type (Figure S5A), suggesting that there is no gross morphological defect of GABA neuromuscular junctions.

We assayed synaptic function from GABA neuromuscular junctions by recording minis in the muscles of *acr-2(n2420gf)* mutants. Our electrophysiological recordings revealed a reduction in GABA neurotransmission in the gain-of-function mutant. However, unlike cholinergic motor neurons, the activity of GABAergic motor neurons was reduced at all concentrations of calcium tested. At 0.5 mM calcium, the mini frequency from GABAergic neurons was only slightly reduced in *acr-2(n2420gf)* animals, 78% compared to the wild type (Figure 5A). But at 2 mM or 5 mM calcium, the mini frequency from GABAergic motor neurons was reduced to about 15% (Figure 5B and 5C). This reduction in neurotransmission from GABAergic motor neurons is consistent with the shrinking behavior observed in these animals.

In addition to a reduction in activity from GABAergic motor neurons, the shrinking behavior could be caused by a reduction in postsynaptic sensitivity to GABA release. We therefore assayed muscle sensitivity to exogenous GABA to determine whether postsynaptic GABA responses are normal in *acr-2(n2420gf)* mutants. We pressure-ejected GABA (100 µM) onto body muscle cells and recorded currents from muscle cells of wild-type or *acr-2(n2420gf)* animals. GABA-evoked outward currents were similar in mean amplitude in the mutant compared to the wild type (Figure S5B), suggesting that GABA receptors are expressed and functional on the membrane of the muscle. To further determine whether the receptors are localized to synapses, we measured GABA-mediated mini amplitudes, which is a measure of the number of receptors activated by the release of GABA from synaptic vesicles at neuromuscular junctions. Mini amplitudes were normal in *acr-2(n2420gf)* mutants (), suggesting that clustering and function of postsynaptic GABA receptors are normal in *acr-2(n2420gf)* mutants. From these results, we inferred that postsynaptic response to GABA is normal, but that neurotransmitter release from the GABAergic motor neuron is impaired in *acr-2* gain-of-function worms. Because *acr-2* is not expressed in GABAergic neurons, depression of neurotransmission in GABAergic neurons is likely to be an indirect effect of hyperactivated cholinergic neurons expressing *acr-2*.

### Extragenic Suppressors of *acr-2(n2420gf)* Identify a Neuronal Acetylcholine Receptor

**Figure 6:**
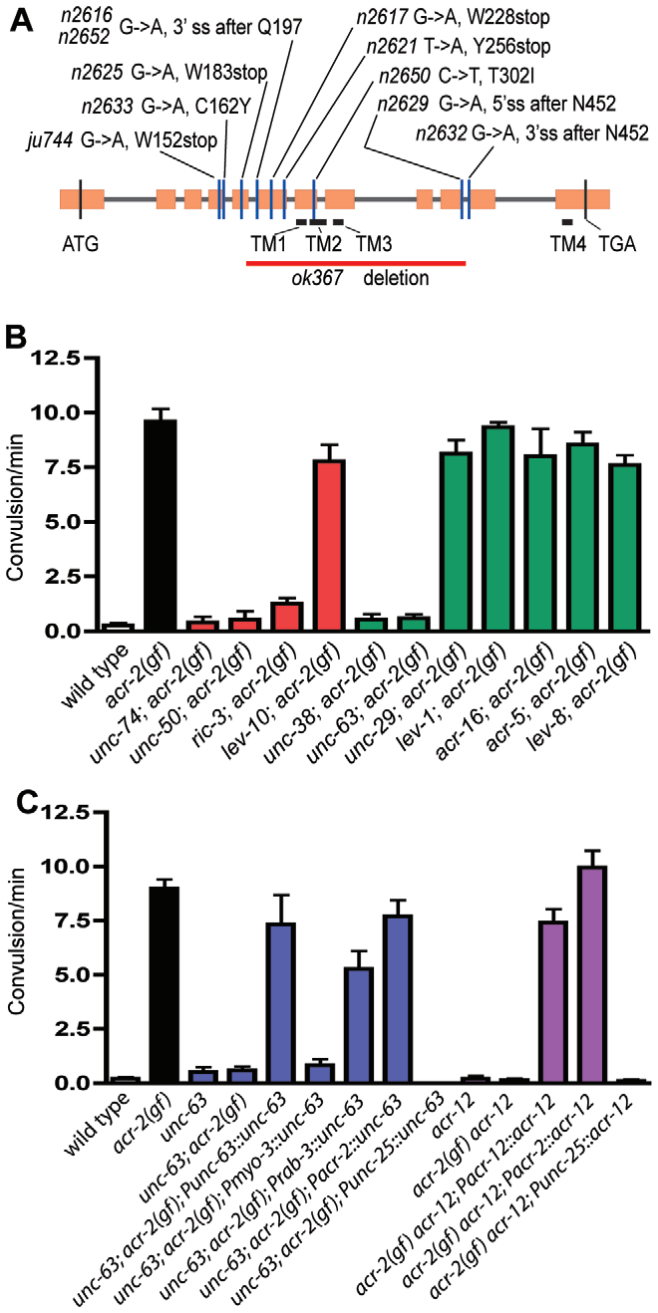
Mutations in acetylcholine receptor subunits suppress convulsions of *acr-2(n2420gf)* mutants. (A) *acr-12* gene structure with the loss-of-function mutations identified as extragenic suppressors of *acr-2(n2420gf)*. *ok367* has a 1,368-bp deletion and is a null allele of *acr-12*. Boxes indicate exons; lines, introns; TM, transmembrane. (B) The convulsions of *acr-2(n2420gf)* mutants are suppressed by loss-of-function mutations in *unc-63*, *unc-38*, *unc-50*, or *unc-74*, but not in *unc-29*, *lev-1*, and several other *acr* genes tested. The alleles used were *unc-63(e384)*, *unc-38(e284)*, *unc-50(n2623)*, *unc-74(n2614)*, *unc-29(x29)*, *lev-1(e211)*, *acr-16(ok789)*, *acr-5(ok180)*, *lev-8(ok1519)*, *ric-3(hm9)*, and *lev-10(x17)*, all of which are null mutations. Red columns represent ancillary proteins, and green columns represent acetylcholine receptor subunits. A minimum of eight to ten animals per genotype were scored. (C) Cell-type–specific transgenic expression of *acr-12* and *unc-63* shows that both are required in the ventral cord cholinergic motor neurons to suppress *acr-2(n2420gf)*. The alleles used were *unc-63(e384)* and *acr-12(ok367)*. *Pmyo-3* promoter drives expression in all body muscles, and *Prab-3* drives expression in all neurons [49]. The *Pacr-2* promoter contains 1.8 kb of *acr-2* upstream sequences driving expression in A and B neurons (Figure 3A), and the *Punc-25* promoter drives expression in the four RME and 19 D neurons [38]. The colors of the columns represent grouping of genotypes. A minimum of eight to ten animals per genotype were scored.

ACR-2 is a non–α-subunit and must interact with other subunits to form a functional receptor. To identify the partners of *acr-2*, we analyzed extragenic suppressor mutations of *acr-2(n2420gf)* (see Materials and Methods). Ten suppressor mutations were linked to chromosome X and fully suppressed convulsions of the *acr-2(n2420gf)* strain. The mutants exhibited no obvious movement defects when separated from the *acr-2* gain-of-function mutation. We mapped one of these mutations to a region between +11.80 and +12.93 (see Materials and Methods). Within this interval is the *acr-12* gene, which encodes an acetylcholine receptor α-subunit that is most similar to the LEV-8 (ACR-13) acetylcholine receptor subunit (Figure 1C). Analysis of the DNA sequence revealed that all ten suppressors contained missense or nonsense mutations in *acr-12* (Figure 6A). An *acr-12(ok367)* deletion mutation that removes a large part of the protein also fully suppressed *acr-2(n2420gf)* convulsion (Figure 6A and 6C). Furthermore, microinjection of a genomic fragment containing the wild-type *acr-12* gene into the suppressed *acr-2(n2420gf) acr-12(ok367)* strain restored the convulsive phenotype in transgenic animals (Figure 6C), thereby confirming the identification of the *acr-12* gene as the suppressor locus.

Nine extragenic suppressor mutations caused sluggish movement when separated from *acr-2(n2420gf)*, and the mutant animals were resistant to the acetylcholine agonist levamisole (Table S1). These mutations fully suppressed the convulsions of *acr-2(n2420gf)* mutants (Figure 6B), but the double mutants remained uncoordinated and resistant to levamisole (Table S1, and unpublished data). Genetic mapping, complementation tests and DNA sequence analyses demonstrated that these nine mutations were alleles of two α-subunits, *unc-38* and *unc-63*, and of two genes required for transport of acetylcholine receptors to the cell surface, *unc-50* and *unc-74* (Table S1) [8],[30] (D. Williams and E. M. Jorgensen, unpublished data).

Multiple lines of evidence indicate that the cellular focus of the ACR-2 and the other acetylcholine receptor subunits is in the cholinergic motor neurons and not in the muscles. First, the other subunit genes that contribute to the levamisole-sensitive receptor in the muscle, *lev-8*, *unc-29*, and *lev-1*, were not identified in the suppressor screen, and mutations in these genes indeed did not suppress the *acr-2(n2420gf)* phenotype in double mutants (Figure 6B). Second, mutations in the nicotine-sensitive muscle receptor *acr-16* did not suppress *acr-2(n2420gf)* (Figure 6B). Third, specific expression of *unc-63* cDNA in cholinergic neurons driven by the 1.8-kb *acr-2* promoter, but not in muscles (by the *myo-3* promoter) or in GABAergic neurons (by the *unc-25* promoter), restored convulsions in *unc-63(lf)*; *acr-2(n2420gf)* double mutants (Figure 6C). Fourth, *acr-12* is expressed in neurons, but not muscles [31],[32]. Specific expression of *acr-12* in the cholinergic motor neurons, but not GABAergic motor neurons, restored convulsions in *acr-2(n2420gf) acr-12(ok367)* double mutants (Figure 6C). Last, we tested mutations in other neuronally expressed acetylcholine receptor subunits, including *acr-5*, *acr-9*, *acr-14*, and *acr-19*, and found that none of them suppressed *acr-2(n2420gf)* (Figure 6B and unpublished data). Together, these results support the conclusion that UNC-38, UNC-63, ACR-12, and ACR-2 are components of a receptor that functions in the cholinergic motor neurons.

### Reconstitution of an ACR-2–Containing Acetylcholine Receptor in *Xenopus* Oocytes

**Figure 7:**
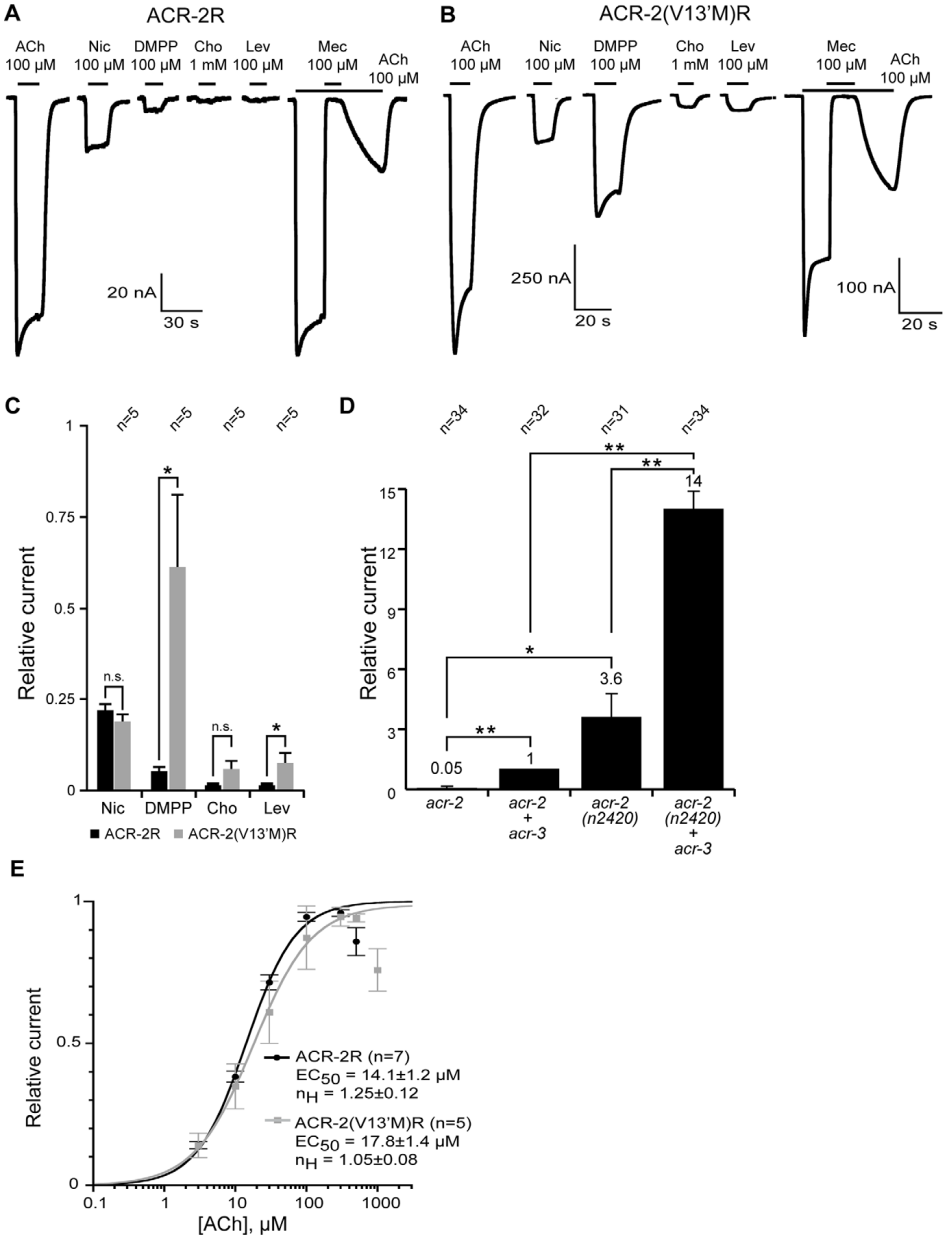
Composition of the ACR-2R receptor reconstituted in *Xenopus* oocytes. (A) The ACR-2R receptor shows high sensitivity to acetylcholine and is insensitive to levamisole or choline. Mecamylamine completely blocks the current. The ACR-2R receptor includes all five subunits. (B) The ACR-2(V13'M)R receptor shows high sensitivity to acetylcholine and an increased sensitivity to DMPP, and weakly responds to levamisole and choline. The ACR-2(V13'M)R receptor includes all five subunits. (C) Quantification of the relative efficacies of cholinergic agonists compared to acetylcholine. Numbers above the bars represent the numbers of oocytes recorded for each condition. Example of traces are shown in (A and B). Replacement of ACR-2(+) by ACR-2(n2420) increased agonist efficacy for DMPP, choline, and levamisole, whereas nicotine efficacy was unchanged. ACh: 100 µM acetylcholine; Nic: 100 µM nicotine; DMPP: 100 µM DMPP; Cho: 1 mM choline; and Lev: 100 µM levamisole. (D) Inclusion of ACR-3 greatly increases the current of the ACR-2R. cRNAs for *acr-12*, *ric-3*, *unc-38*, *unc-50*, *unc-63*, and *unc-74* were coinjected for each condition. Average peak current for *acr-2 *+ *acr-3* coinjection was 111±68 nA (*n* = 34). (E) Dose–response curves for acetylcholine action on the ACR-2 and ACR-2(V13'M) receptors show that EC50 is comparable. All recordings were made with 1 mM external CaCl_2_. Error bars are standard errors of the mean. Asterisks indicate that data are significantly different at *p*<0.05 (*) or *p*<0.01 (**). ACh: acetylcholine; Nic: nicotine; DMPP: 1,1-dimethyl-4-phenylpiperazinium; Cho: choline; Lev: levamisole; Mec: mecamylamine.

To further verify the subunit composition of the ACR-2–containing receptor (referred to as ACR-2R) and characterize its pharmacology, we performed reconstitution experiments using *Xenopus* oocytes. Previous attempts to reconstitute *C. elegans* levamisole-sensitive acetylcholine receptors in *Xenopus* oocytes demonstrated that the requirements for functional expression in vitro recapitulate genetic requirements in vivo [13]. Specifically, the ancillary proteins UNC-50 and UNC-74, and to a lesser extent RIC-3, which are involved in the assembly and trafficking of levamisole-sensitive acetylcholine receptors in worms [33], are required for function of levamisole-sensitive acetylcholine receptors in oocytes. The finding that loss-of-function mutations in *unc-50* and *unc-74* suppress *acr-2(n2420gf)* suggests that these two ancillary proteins also function in ACR-2R assembly and trafficking. *ric-3* animals were not identified in the *acr-2(n2420gf)* suppressor screen, likely because these animals are severely uncoordinated and unhealthy. We therefore constructed *ric-3*; *acr-2(n2420gf)* double mutants and found that the convulsion frequency was dramatically reduced (Figure 6B), indicating a requirement of *ric-3* for *acr-2(n2420gf)* function. Consequently, we coinjected cRNAs for *acr-2*, *acr-12*, *unc-38*, and *unc-63*, together with *unc-50*, *unc-74*, and *ric-3* cRNAs at equal molar ratios (see Materials and Methods). This experiment yielded little or no current (Figure 7D), suggesting that a factor was missing. Since the closely related acetylcholine receptor subunit *acr-3* is part of the *acr-2* operon (Figure 1B and 1C), it is likely that the ACR-2 and ACR-3 subunits are coexpressed in the same cells. When the *acr-3* cRNA was added to the previous injection mix, robust expression of an acetylcholine-gated ion channel was observed (Figure 7A and 7D). Pharmacological characterization demonstrated that the ACR-2R channel was weakly activated by nicotine and DMPP, and almost completely insensitive to levamisole or choline, and was efficiently blocked by mecamylamine (Figure 7A and 7C). The estimated median effective concentration (EC_50_) of the ACR-2R receptor was 14.1±1.2 µM, and the Hill coefficient was 1.25±0.12 (Figure 7E).

To further analyze the impact of the *acr-2(n2420gf)* mutation on receptor physiology, we replaced the wild-type cRNA of *acr-2* with a cRNA carrying the *n2420* mutation, and analyzed the mutant receptor (referred as ACR-2(V13′M)R). Introduction of this point mutation caused a 14-fold increase in current compared to the wild-type ACR-2 subunit (Figure 7D). The pharmacological profile of the ACR-2(V13′M) receptor was also modified: 1) response to 100 µM DMPP was strongly increased, and 2) choline and levamisole caused modest receptor activation (Figure 7B and 7C). However, the acetylcholine EC_50_ was not significantly changed (17.8±1.4 µM; Figure 7E), and no leak current could be recorded. The ACR-2(V13′M) receptor remained fully blocked by mecamylamine, in agreement with the suppression of convulsions of *acr-2(n2420gf)* by this drug (Figure 2A).

*acr-3* was required in oocytes for expression of ACR-2R receptors, but *acr-3* was not identified as an extragenic suppressor of *acr-2(n2420gf)*. Moreover, a loss-of-function mutation in *acr-3* did not affect the convulsion behavior caused by transgenic expression of *acr-2(V13′M)* (Figure S6). Hence, we analyzed the properties of a putative ACR-2(V13′M) receptor missing ACR-3. Removing *acr-3* cRNA from the injection mix only partially reduced the average current size, which remained almost 4-fold higher than what we observed with the full complement of wild-type subunits (Figure 7D). This finding likely explains why eliminating *acr-3* in an *acr-2(n2420gf)* gain-of-function background would not lower the convulsion phenotype enough for it to be identified in our suppressor screen. In summary, our oocyte reconstitution studies identified the complete molecular composition of ACR-2 channel and demonstrated that it is a bona fide acetylcholine receptor.

## Discussion

The vast number and overlapping expression of acetylcholine receptor subunits make it challenging to decipher the in vivo composition of functional acetylcholine channels. By analyzing suppressor mutations of an activated ACR-2 acetylcholine receptor, we were able to define the components of this neuronal acetylcholine receptor. Our studies reveal four aspects of acetylcholine receptor function in *C. elegans*. First, pharmacology and function of acetylcholine receptors is the result of combinatorial subunit assembly in neurons and muscles. Second, a gain-of-function mutation in the pore domain of the neuronal receptor affects the pharmacology of the channel. Third, the ACR-2 neuronal receptor maintains motor neuron excitability in locomotion. Fourth, the physiological consequences of the gain-of-function ACR-2 receptor have revealed an unexpected inhibitory relationship between the cholinergic and GABAergic motor neurons within the motor circuit.

### Composition and Characteristics of the ACR-2 Acetylcholine Receptor

The ACR-2–containing acetylcholine receptor in neurons is closely related to the levamisole-sensitive acetylcholine receptor that functions in *C. elegans* body muscle. Both receptors contain five distinct subunits, including three α- and two non–α-subunits. The UNC-38 and UNC-63 α-subunits are common to both receptors, yet the pharmacological profiles of the two receptors are very distinct (Table 1). ACR-2R receptors are slightly more sensitive to acetylcholine than levamisole-sensitive receptors, with an EC_50_ of 14 µM as compared to 26 µM, respectively (this study and [13]). Strikingly, levamisole has no effect on the ACR-2R neuronal receptor but potently activates the levamisole-sensitive muscle receptor. Nicotine weakly activates ACR-2R receptor but inhibits the levamisole receptor (this study and [13]). In ionotropic acetylcholine receptors, agonist binding sites are formed at the interface between the (+) side of an α-subunit and the (−) side of the adjacent subunit [4],[5]. Binding sites for noncompetitive agonists and antagonists have also been identified at the non-α(+)/α(−) subunit interface [34]. If UNC-38 and UNC-63 are not adjacent within receptor pentamers, the unique subunits (Table 1) will modify the complementary surface of each binding site and change the binding pocket and transduction residues for these drugs.

**Table 1:** Comparison of ACR-2R and levamisole-sensitive receptors.

	ACR-2R (Neuronal)	Levamisole Receptor (Muscle)
Alpha	UNC-38	UNC-38
Alpha	UNC-63	UNC-63
Alpha	ACR-12	LEV-8
Non-alpha	ACR-2	UNC-29
Non-alpha	ACR-3	LEV-1
ACh EC_50_	14 µM	26 µM
Levamisole	No effect	Agonist
Nicotine	Weak agonist	Antagonist

Ach, acetylcholine; EC_50_, median effective concentration.

### Effects of V13′M Mutation on ACR-2R Channel Activity

The gain-of-function mutation in *acr-2* changes a valine to a methionine at the 13′ position of the pore-forming transmembrane domain of the ACR-2 subunit. The 13′ position is in the upper half of the lumen and faces the pore [20]. A valine residue at this position is highly conserved in acetylcholine receptors, suggesting that it is important for proper receptor function. A V13′M mutation in the β1-subunit of the human muscle acetylcholine receptor causes myasthenia gravis [22]. When expressed in HEK cells, the receptors containing the β1(V13′M)subunit exhibited higher acetylcholine affinity than the wild-type receptor [22]. Single-channel recording indicated that the mutant channel had longer open times and spontaneous openings. Patients with the hyperactive receptor displayed progressive degeneration of muscle end-plates that characterizes the myasthenic syndrome. The importance of the valine residue at position 13′ was also investigated in the chick α7-subunit. This subunit forms a homomeric nicotinic receptor that can be efficiently expressed in *Xenopus* oocytes. Mutating this valine into a threonine causes an almost 10-fold increase in the mean current amplitude and a 100-fold increase in the acetylcholine affinity [35]. Since this mutation affects a residue facing the pore lumen, increased current amplitude could arise simply from changed channel conductance. However, the single V13′T point mutation in the α7-subunit has pleiotropic effects. Specifically, multiple channel conductances were identified; moreover, the competitive antagonist DHβE was converted into a partial agonist [35]. These effects were interpreted as a change in the allosteric states of the channel as it transitions to the desensitized state [36]. The V13′M mutation in the ACR-2 subunit causes similar defects. Recording of ACR-2R expressed in *Xenopus* oocytes shows that mutating this valine caused a dramatic increase in currents, similar to the chick α7-subunit. ACR-2R receptor pharmacology is also changed; DMPP elicits larger currents from the mutant channel than the wild-type channel, whereas activation by nicotine is unaffected. These phenotypes cannot be explained by a simple change in efficiency of receptor assembly or increased channel conductance but rather suggest that changes to the pore affect dynamic transitions throughout the receptor.

Characterization of *acr-2(n2420gf)* mutant animals suggests that the ACR-2(V13″M) subunit generates a hyperactive channel in vivo. Worms expressing the ACR-2(V13′M) subunit exhibit spontaneous convulsions, which can be reversed by the channel-blocker mecamylamine. In addition, convulsions can be suppressed by null mutations in genes encoding any of the subunits of the receptor except the *acr-3* gene. The nonessential role of ACR-3 can be explained by the observation that a functional channel is formed in the absence of ACR-3 when the ACR–2 subunit contains the V13′M mutation. Although the mutant ACR-2(V13′M) channel is less active if it lacks the ACR-3 subunit, it is still almost four times more active than the wild-type receptor. The valine at the 13′ position of the pore in the chick α7 acetylcholine receptor limits calcium influx [20],[37]. Our recordings from the *acr-2(n2420gf)* mutant animals also show that neuronal activity involving ACR-2(V13′M)R is hypersensitive to calcium levels. Thus, in vivo, the ACR-2(V13′M) gain-of-function channel might result in increased excitability of the neurons and increased calcium influx, which could have broader effects because of the action of calcium as a second messenger.

### ACR-2 Channel Functions to Maintain the Excitability of the Cholinergic Motor Neurons

ACR-2 is expressed and functions in the ventral cord cholinergic motor neurons that provide the major excitatory inputs to the body muscles involved in locomotion. Of these motor neurons, VA and VB innervate the ventral muscles, and DA and DB innervate the dorsal muscles [15]. These motor neurons are required for the sinusoidal posture and locomotion of the worm. Animals lacking ACR-2 are still capable of locomotion, but they move more slowly. Our electrophysiological recordings from muscles demonstrate that the ACR-2 receptor is required to maintain normal levels of excitation in the cholinergic motor neurons. The cholinergic motor neurons showed reduced neurotransmitter release in *acr-2(lf)* animals, whereas these motor neurons in *acr-2(n2420gf)* animals displayed normal morphology and increased neurotransmitter release. ACR-2 could maintain the activity state of these neurons by regulating presynaptic release directly, perhaps as an autoreceptor, or indirectly through other pathways.

Our data further provide functional evidence for inputs from cholinergic motor neurons into GABAergic motor neurons. The GABAergic neurons have processes adjacent to acetylcholine neuromuscular junctions and based on electron micrograph reconstructions of the nervous system appear to receive input from cholinergic motor neurons at dyadic synapses [15]. Our data are consistent with a stimulatory input from cholinergic neurons to GABAergic neurons. In *acr-2* loss-of-function mutants, a reduction in cholinergic motor neuron activity is coupled with a reduction in GABAergic motor neuron activity.

The gain-of-function mutation in *acr-2* also exhibits nonautonomous effects on the GABAergic motor neurons. The *acr-2(n2420gf)* mutant was originally identified because it exhibited a spontaneous shrinking behavior. Shrinking typifies mutants with defects in GABA transmission. Consistent with this phenotype, physiological recordings from dissected animals demonstrated that GABA transmission was greatly reduced in *acr-2(n2420gf)* mutants. However, other mutants that eliminate GABA function, such as mutations in the biosynthetic enzyme for GABA or in the GABA receptors do not exhibit spontaneous hypercontractions [38],[39]. In addition, other mutations with hyperactivation of the cholinergic motor neurons, such as mutations in Goα or in the calcium-activated potassium channel [24],[40], do not show the convulsive shrinking behavior. The convulsive nature of the *acr-2(n2420gf)* mutant rather relies on the simultaneous activation of the cholinergic motor neurons and the nonautonomous suppression of activity in the GABAergic motor neurons. In these mutants, homeostatic mechanisms within the motor circuit do not seem to compensate for the imbalance in network activity; in fact, the imbalance in excitation and inhibition is most severe at physiological levels of calcium. The convulsive behaviors of *acr-2(n2420gf)* bear similarities to the neurological features underlying some forms of epilepsy [41]. For example, genetic mutations in nicotinic acetylcholine receptors have been linked to frontal lobe epilepsy [42]. In the future, it will be interesting to determine the mode of ACR-2–mediated neurotransmission and how changes in motor circuit properties suppress or contribute to such imbalances.

## Materials and Methods

### Genetics

All *C. elegans* strains were grown at 20°C as described [43]. The wild-type strain N2 was mutagenized with EMS following standard procedures [43]. The *n2420* mutation was isolated based on its shrinker behavior from among the F2 progeny of animals carrying approximately 6,000 mutagenized haploid genomes. The *n2420* mutation was backcrossed against N2 multiple times. It was mapped to the X chromosome by linkage to *lon-2*; also, *n2420* males showed spontaneous shrinking behavior. Further three-factor mapping placed *n2420* between *dpy-8* and *unc-6*: from *n2420/dpy-8 unc-6* heterozygotes, 18/19 Dpy non-Unc segregated *n2420*, and 1/18 Unc non-Dpy segregated *n2420*. We tested two deficiencies *uDf1* and *stDf1* that remove the region containing *acr-2* and observed that *n2420/Df* animals exhibited wild-type movement as did *Df/+* animals.

Suppressors of *n2420* were isolated as following: ten EMS-mutagenized *n2420* L4 P0 animals were placed on a large NGM plate and were transferred to fresh plates daily for 2 d. Young adult F2 animals were collected from each P0 plate and placed away from the bacteria food on a new plate. After 1 h, worms that had crawled into the food were collected. Only one to two such animals per plate were saved to ensure independence of isolates. We screened an estimated 120,000 mutagenized haploid genomes. Fifty-three suppressor mutants were backcrossed with N2. A list of the strains containing suppressor mutations is in Table S1. We identified those that did not segregate the *n2420* mutant phenotype after backcrossing as presumptive intragenic mutations, for which we determined DNA sequences of *acr-2* locus. Extragenic suppressor mutations segregated *n2420*-like animals and were grouped into levamisole-resistant or levamisole-sensitive classes. Complementation tests with known levamisole-resistant mutants were performed using standard procedures, and DNA sequence determination of the suppressor mutants subsequently confirmed gene identities. The *acr-12(n2616)* mutation was mapped between X:11.80 (*pkP6133*) and X:12.93 (*pkP6122*) using single-nucleotide polymorphisms between the N2 strain and the Hawaiian strain CB4856 [44],[45]. All *acr-12* mutations were confirmed by DNA sequence determination. Other double mutants were constructed using standard procedures, and genotypes were confirmed by allele sequence determination. Information about these strains is shown in Table S2.

### Molecular Biology

General molecular biology was performed according to Sambrook et al. [46]. A pJB8-based cosmid library [47] was used in the initial germline transformation rescue of the *acr-2(n2420gf)* phenotype. Subclones pSC175, pSC176, and pSC178 were generated from the rescuing cosmid C46C10 clone. Cosmid and plasmid DNAs were injected at 10 ng/ml and 50 ng/ml, respectively, using pRF4 as a coinjection marker following standard procedures [48]. Multiple independent lines were examined for rescue of the convulsion phenotype. For mutation sequence determination, pairs of primers were used to amplify all exons and exon–intron boundaries. *acr-2* cDNAs were isolated by screening a mixed-stage cDNA library prepared by P. Okkema, using *acr-2* genomic DNA as probe. Four independent clones were isolated from 2×10^6^ plaques. Three had similarly sized inserts and identical end sequences. Full sequences of the cDNA 21A clone were determined, which confirmed the predicted gene structure of *acr-2*.

Transcriptional *acr-2* promoter-driven GFP (pSC205) or mCherry (pCZGY847) constructs were made using 3.5 kb or 1.8 kb of *acr-2* 5′ upstream sequences, respectively. The 3.5-kb promoter also included the entire upstream gene F38B6.1 and portion of F38B6.2; the 1.8-kb promoter included only the promoter region of *acr-2*. *Punc-25-acr-2* (pSC374) was constructed by replacing the *acr-2* promoter with 1-kb *unc-25* promoter. *Pacr-12::acr-12* transgenes was generated using PCR-amplified *acr-12* genomic DNA that included 1.4 kb of 5′ upstream sequences, the entire coding region, and 0.9 kb of 3′ downstream sequences. *unc-63* cDNA was subcloned from pAF55 (*Prab-3::unc-*63) [49]. *Punc-25-acr-12* (pCZGY745), *Pacr-2-acr-12* (pCZGY744), *Punc-25-unc-63(cDNA)* (pCZGY745), and *Pacr-2-unc-63(cDNA)* (pCZGY744) were constructed using the Gateway cloning technology (Invitrogen) (Table S2). The sequences of resulting DNA clones were confirmed. Transgenic lines were generated using either *plin-15(+)*, pRF4, or *Pttx-3*-XFP as coinjection markers (Table S2). Integration of extrachromosomal arrays was preformed following Trimethyl Psoralen-UV mutagenesis.

### Quantification of Convulsion Rates

Ten to 20 L4 larvae were placed on freshly seeded NGM plates. The following day, young adults were transferred to fresh plates and recorded by video for 90 s, five frames per second. Videos were scored by observers blind to genotype. A “convulsion” was defined as an event involving the nose of the worm moved backwards without the tail of the worm moving. For each strain, video observation was performed on worms from at least two independent experiments.

### Pharmacology Analysis

All drug manipulations were performed according to published procedures [7],[8],[21]. Drugs were purchased from Sigma-Aldrich. For levamisole and aldicarb assays, 1-d-old adult hermaphrodites were placed on plates containing the drug of chosen concentration, and the effects on animal movement were observed at 15- to 30-min intervals. Animals were scored as paralyzed when no body movements were observed in response to poking. In mecamylamine tests, the effects of the drug on *acr-2(n2420gf)* animals were first assessed using a concentration series from 50 µM to 400 µM, and the behavior of *acr-2(n2420gf)* animals was suppressed to nearly wild type after 5 h on plates containing 100 µM to 400 µM mecamylamine. Quantification of the convulsion rate was performed on 1-d-old adult hermaphrodites. Animals were first placed on seeded plates with no drug, and the convulsion rate was recorded by video as above to set time 0. The animals were then transferred to seeded plates containing 100 µM mecamylamine, and the convulsion rate recorded every 60 min for 3 h. Animals were then transferred to plates containing no drug and recorded by video at 30 min afterwards.

### Worm-Tracking Assay

Worm-tracking experiments were performed according to [50]. Standard NGM plates were prepared with the addition of 0.01% bromophenol blue (Sigma-Aldrich) and were allowed to cool for at least 5 h. Plates were then spread with 240 µl of 2% HB101 bacteria in M9 medium and were incubated overnight at room temperature. The following day, five gravid worms were placed on each plate in a 5-µl drop of M9 medium. Assays were recorded at a frequency of 1 frame/s for 10 min, starting when the drops of M9 had absorbed. Video images were analyzed using ImageJ software (NIH).

### Electrophysiological Studies

Electrophysiological methods were adapted from previous studies [28],[51]. Adult nematodes were glued (Histoacryl Blue, B. Braun) along the dorsal side of the body to the surface of a plastic coverslip. A sharpened tungsten rod (A-M Systems) was used to perform a lateral incision and to remove the viscera. The cuticle flap was glued back to expose the ventral medial body wall muscles, and the preparation was treated by collagenase type IV for 20 s at a concentration of 0.5 mg/ml.

For Figures 4, 5A, 5B, S2, and S4, membrane currents were recorded in the whole-cell configuration using an EPC-10 patch-clamp amplifier (HEKA). Acquisition and command voltage were controlled using the HEKA Patchmaster software. For Figures 5C and S3, membrane currents were recorded using a RK-400 patch-clamp amplifier (Bio-Logic). Acquisition and command voltage were controlled using the pClamp9 software (Axon Instruments) driving a 1322A Digidata (Axon Instruments). Data were analyzed and graphed using Mini Analysis (Synaptosoft) and Microcal Origin software (Microcal Software). The resistance of recording pipettes was within 3–4.5 MΩ. Capacitance, resistance, and leak current were not compensated. All experiments were performed at room temperature.

The bath solution contained 150 mM NaCl, 5 mM KCl, 1 mM MgCl_2_, 10 mM glucose, 15 mM HEPES, and sucrose to 340 mOsm (pH 7.35). External CaCl_2_ concentration was 0.5, or 2 or 5 mM, as indicated in each figure. For the 0.5 mM CaCl_2_ solution, the concentration of MgCl_2_ was increased to 4 mM in order to help stabilize the membrane [52]. The pipette solution contained 125 mM K gluconate, 20 mM KOH, 10 mM hepes, 1 mM MgATP, 3 mM NaATP, 5 mM EGTA, 15 mM KCl, and sucrose to 335 mOsm (pH 7.2). GABA was diluted to 0.1 mM in the bath solution containing 2 mM CaCl_2_ and was pressure-ejected in the vicinity of muscle cells. All chemicals were obtained from Sigma-Aldrich.

### Electrophysiological Studies of *X. laevis* Oocytes

*X. laevis* oocytes were prepared, injected, voltage-clamped, and superfused according to the procedure described in [13]. Each set of recordings was done on the same day, 2 or 3 d after the cRNA injections. Dose-response experiments were performed as described in [13]. Values obtained at 500 µM and 1 mM were excluded from the fit because of the open-channel block observed at high acetylcholine concentrations. RNA isolation was performed as described in [13]. cDNAs were obtained by reverse-transcription PCR using the following primer combinations.

*acr-2(+)* and *acr-2(n2420gf)*: oTB429 5′-AAACTCGAGatgaagaagacggtcaaaat-3′ and oTB430 5′-TTTGGGCCCttaagaatacatatcagac-3′

*acr-3*: oTB439 5′-AAACTCGAGatgcagaaaatatggttatt-3′ and oTB440-5′-TTTGGGCCCtcatgaattcaacatttc-3′;

*acr-12*: oTB431 5′-AAACTCGAGatgctctataaaaaacg-3′ and oTB432-5′-TTTGGGCCCtcacttcaagttccatgaac-3′.

PCR fragments were digested with XhoI and Bsp120I restriction enzymes and cloned into pTB207, an expression vector for in vitro transcription that contains the 3′ UTR of the *Xenopus laevis* β-globin gene. The resulting plasmid clones are pTB244 *acr-2*, pTB245 *acr-2(n2420gf)*, pTB246 *acr-12*, and pTB247 *acr-3*. In addition, we used the following clones described in [13]: pTB211 *unc-38*, p+TB212 *unc-63*, pTB215 *ric-3*, pTB216 *unc-74*, and pTB217 *unc-50*.

cRNA was synthesized in vitro from linearized plasmid DNA templates using the mMessage mMachine T7 transcription kit (Ambion). Lithium chloride–precipitated cRNA was resuspended in RNAse-free water and stored at −80°C.

Acetylcholine chloride (ACh), (−)-nicotine hydrogen tartrate (Nic), 1,1-dimethyl-4-phenylpiperazinium iodide (DMPP), choline bitartrate (Cho), (−)-tetramisole hydrochloride (levamisole, Lev), mecamylamine hydrochloride (Mec) were purchased from Sigma-Aldrich.

## Supporting Information

Protocol S1 **Supplementary materials and methods.**
(0.02 MB DOC)

Figure S1 **Sequence alignment of ACR-2 with other non-α acetylcholine receptor subunits.** Green letter marks the Val309 that is mutated to Met in *acr-2(n2420gf)*. Purple letters mark the amino acid positions that are mutated to stop codons in several intragenic suppressors of *acr-2(n2420gf)*. We used *n2595* as a null allele of *acr-2*. Red letters mark the amino acid substitutions in other intragenic suppressors of *acr-2(n2420gf)*. Details of the nucleotide and amino acid changes in these *acr-2* alleles are shown in Table S1. Ce, *C. elegans*; Hu, human; TOR, Torpedo. GenBank numbers are Hu_alpha3 (CAD88991), TOR_alpha (AAA96705), TOR_gamma (AAR29362), and Ce_ACR-2 (AAK71377).
(0.36 MB PDF)

Figure S2 **Mini amplitude is not altered in** ***acr-2(n2595 n2420)*** **.** Amplitude histograms (top) and cumulative amplitude distribution (bottom) of acetylcholine (left) and GABA mini (right) from the wild type (*n* = 5) and *acr-2(n2595 n2420)* (*n* = 9) in 2 mM external CaCl_2_.
(0.21 MB PDF)

Figure S3 **Acetylcholine neurotransmission is reduced in** ***acr-2(ok1887)*** **mutants.** (A) Representative traces of minis recorded at two holding potentials, −60 and −10 mV, on body muscle cells from wild-type and *acr-2(ok1887)* worms in 2 mM external CaCl_2_. (B) Acetylcholine mini frequencies recorded in 2 mM external CaCl_2_ from the wild type (18.6 events/s±2.3 standard error of the mean [SEM], *n* = 8) and *acr-2(ok1887)* (11.6 events/s±1.4 SEM, *n* = 12) are significantly different (*p = 0.0144.). GABA mini frequencies recorded from the wild type (9.8 events/s±2.3 SEM, *n* = 8) and *acr-2(ok1887)* (6.6 events/s±1.9 SEM, *n* = 12) are not significantly different (*p* = 0.3003). Data were analyzed using a two-tailed unpaired *t*-test. (C) Amplitude histograms (top) and cumulative amplitude distribution (bottom) of acetylcholine (left) and GABA mini (right) from wild-type (*n* = 8) and *acr-2(ok1887)* (*n* = 12) worms in 2 mM external CaCl_2_.
(0.98 MB PDF)

Figure S4 **Cholinergic motor neuron morphology and synapses are not altered in** ***acr-2(n2420gf)*** **mutants.** (A) Cholinergic A- and B-type motor neurons visualized with *Pacr-2-GFP* (*juIs14*) show normal position and morphology in *acr-2*(*n2420gf*) animals. Scale bar indicates 20 µm. (B) Pattern of DA and DB synapses visualized by *Pacr-2-SNB-1::GFP* (*juIs20*) is similar in *acr-2(n2420gf)* and wild-type animals. Scale bar indicates 10 µm. (C) Quantification of the SNB-1::GFP puncta number in a segment of the dorsal cord. N indicates the number of animals for each genotype. Statistics: unpaired Student *t*-test; error bars indicate the standard error of the mean; n.s., not significant. See Protocol S1 for image collection and analysis.
(0.43 MB PDF)

Figure S5 **GABA receptors are not altered in** ***acr-2(n2420gf)*** **mutants.** (A) Distribution of GABA synapses. Left panel shows the expression pattern of GFP-tagged synaptobrevin (*Punc-25:GFP:* synaptobrevin) in the dorsal cord of young adult wild-type and *acr-2(n2420gf)* animals, just posterior to the vulva. Quantification of the GFP puncta number is shown on the right. For wild type: 1.9 puncta/10 µm ±0.1 SEM, *n* = 8 and for *acr-2*(*n2420gf*): 1.9 puncta/10 µm ±0.1 SEM, *n* = 11. Scale bars indicate 10 µm. Data were analyzed using two-tailed unpaired *t*-tests. See Protocol S1 for image collection and analysis. (B) Amplitude histograms (top) and cumulative amplitude distribution (bottom) of acetylcholine (left) and GABA minis (right) from the wild type (*n* = 10) and *acr-2(n2420gf)* (*n* = 14) in 2 mM external CaCl_2_. (C) Representative traces and mean amplitude of currents evoked by 0.1 mM pressure-ejected GABA on muscle cells from the wild type (513.5 pA ±52.5 SEM, *n* = 6) and *acr-2(n2420gf)* (597.2 pA ±53.7 SEM, *n* = 14) in 2 mM external CaCl_2_. Mean amplitudes were compared using a two-tailed unpaired *t*-test.
(0.52 MB PDF)

Figure S6 **Loss-of-function** ***acr-3*** **mutations do not suppress convulsions in transgenic animals expressing** ***acr-2(n2420gf)*** **.** *n* = 10 animals per genotype. ns, not significant. See Protocol S1 for transgene construction.
(0.35 MB PDF)

Table S1 **Summary of suppressor mutations.**
(0.04 MB DOC)

Table S2 **Strains and genotypes.**
(0.03 MB DOC)

Video S1 ***acr-2(n2420gf)*** **convulsion.**
(2.44 MB MOV)

Video S2 **Suppression of the convulsion defects of** ***acr-2(n2420gf)*** **by mecamylamine.**
(0.87 MB MOV)

## Abbreviations

EC_50_: median effective concentration
mini: miniature postsynaptic current
