# Supplementary material for: A Neuronal Acetylcholine Receptor Regulates the Balance of Muscle Excitation and Inhibition in Caenorhabditis elegans
Source: PLoS Biol. 2009 Dec 22;7(12):e1000265. doi: 10.1371/journal.pbio.1000265 (PMC2787625; doi:10.1371/journal.pbio.1000265)
Supplement: Protocol S1 — Supplementary materials and methods. (0.02 MB DOC) [file pbio.1000265.s001.doc]

**Supplementary Materials and Methods**

**Quantitative Fluorescence Microscopy**

Images were collected using a 63x objective on a Zeiss Pascal or LSM510 Confocal microscope. Animals were immobilized with 2% phenoxypropanol. All images were taken with the same settings on the same day after the laser was allowed to stabilize for 30 min. For strains expressing synaptobrevin:GFP all images were taken of the same region of the dorsal cord with the animals facing the dorsal side.

**Transgenic *acr-2(n2420gf)* animals**

The genomic region of *acr-2(n2420gf)* containing the 1,904 bp sequences upstream of the ATG, 6,451 bp of coding sequences and 993 bp of 3’ UTR was amplified by PCR using primers 5’-ATTGTCGGCCCGTTTTGTTTTC and 5’-GACACTGAATCGCACTTGCTTCC. The PCR product was injected into N2 animals at 20 ng/μl along with 50 ng/μl of *Pttx-3-GFP* as a co-injection marker and 30 ng/μl of pBlueScript as carrier DNAs. Multiple transgenic lines were obtained, and all displayed convulsions similar to those of *acr-2(n2420gf)* mutants. The extrachromosomal array *juEx2033* was crossed into the *acr-3(ok2049)* mutants.
