# Supplementary material for: A Neuronal Acetylcholine Receptor Regulates the Balance of Muscle Excitation and Inhibition in Caenorhabditis elegans
Source: PLoS Biol. 2009 Dec 22;7(12):e1000265. doi: 10.1371/journal.pbio.1000265 (PMC2787625; doi:10.1371/journal.pbio.1000265)
Supplement: Figure S1 — Sequence alignment of ACR-2 with other non-α acetylcholine receptor subunits. Green letter marks the Val309 that is mutated to Met in acr-2(n2420gf). Purple letters mark the amino acid positions that are mutated to stop codons in several intragenic suppressors of acr-2(n2420gf). We used n2595 as a null allele of acr-2. Red letters mark the amino acid substitutions in other intragenic suppressors of acr-2(n2420gf). Details of the nucleotide and amino acid changes in these acr-2 alleles are shown in Table S1. Ce, C. elegans; Hu, human; TOR, Torpedo. GenBank numbers are Hu_alpha3 (CAD88991), TOR_alpha (AAA96705), TOR_gamma (AAR29362), and Ce_ACR-2 (AAK71377). (0.36 MB PDF) [file pbio.1000265.s002.pdf]

```
Ce_ACR-2     MKKTVKILLILITVFLKVHCNGGHDDEAADFLSHTNIDDPNNSSDPNKNSDQGDTMGEDE   60
HU_alpha3    ---------MGSGPLSLPLALSPPRLLLLLLLSLLPVARAS-----------------EAE   35
TOR_alpha    -----------------MILCSYWHVGLVLLLFSCCGLVLGS-----------------EHE   28
TOR_gamma    -------------------------MVLTLLLIICLALEVRS-----------------NEE   20
                                           ::    :   .                 : *

                   F
Ce_ACR-2     DRLVIDLFREYNFLIRPVKNVSSPPVVVDFGVAMILLINVDEKNQILQTNVWLTMKWNDF   120
HU_alpha3    HRLFERLFEDYNEIIRPVANVS-DPVIIHFEVSMSQLVKVDEVNQIMETNLWLKQIWNDY   94
TOR_alpha    TRLVANLLENYNKVIRPVEHHT-HFVDITVGLQLIQLINVDEVNQIVETNVRLRQQWIDV   87
TOR_gamma    GRLIEKLLGDYDKRIKPAKTLD-HVIDVTLKLTLTNLISLNEKEEALTTNVWIEIQWNDY   79
              **.   *: :*:   *:*.        : :  . : :  *:.::* :: : **: :    * *
                                                                (n2595)
                  L                      V          F              X  SS
Ce_ACR-2     QLAWNPAEYGNISNLHVPSDRVWLPDIVLFNNADGNYEVSFKSNVFVDHHGDVTWVPPAM   180
HU_alpha3    KLKWNPSDYGGAEFMRVPAQKIWKPDIVLYNNAVGDFQVDDKTKALLKYTGEVTWIPPAI   154
TOR_alpha    RLRWNPADYGGIKKIRLPSDDVWLPDLVLYNNADGDFAIVHMTKLLLDYTGKIMWTPPAI   147
TOR_gamma    RLSWNTSEYEGIDLVRIPSELLWLPDVVLENNVDGQFEVAYYANVLVYNDGSMYWLPPAI   139
             :* **.::*  .  . :::*:: :* **:** **. *:: :    :: ::    *.: * ***:

                                     LX    K                           N F  E
Ce_ACR-2     FKSSCRIDVEWFPFDEQCCTLVFGSWTYNSEEVRLHWYNN------IQAVQLHDYSYSGI   234
HU_alpha3    FKSSCKIDVTYFPFDYQNCTMKFGSWSYDKAKIDLVLIG--------SSMNLKDYWESGE   206
TOR_alpha    FKSYCEIIVTHFPFDQQNCTMKLGIWTYDGTKVSISPES--------DRPDLSTFMESGE   199
TOR_gamma    YRSTCPIAVTYFPFDWQNCSLVFRSQTYNAHEVNLQLSAEEGEVVEWIHIDPEDFTENGE   199
             ::* * * *   **** * *:: :    :*:   :: :                :   :   .*

                   S
Ce_ACR-2     WDVIDVPG-----QLVHKPDLKENKMVFNVVIRRKTLFYTVILIIPTVLMAFLSVMAFYL   289
HU_alpha3    WAIIKAPGYKHDIKYNCCEEI-YPDITYSLYIRRLPLFYTINLIIPCLLISFLTVLVFYL   265
TOR_alpha    WVMKDYRGWKHWVYYTCCPDTPYLDITYHFIMQRIPLYFVVNVIIPCLLFSFLTVLVFYL   259
TOR_gamma    WTIRHRPAKKNYNWQLTKDDIDFQEIIFFLIIQRKPLFYIINIIAPCVLISSLVVLVYFL   259
             * :  .   .           :       .: :  . ::* .*:: : :* * :*:: * *:.::*
                                                        _______________________
                                                                 TM1
                         (n2420)
                            M*          I     F
Ce_ACR-2     PVDSG-EKVSLTISLLLALVVFLLLVSKILPPTSN-IPLMGKYLLLAFVLNITAVVGTVV   347
HU_alpha3    PSDCG-EKVTLCISVLLSLTVFLLVITETIPSTSLVIPLIGEYLLFTMIFVTLSIVITVF   324
TOR_alpha    PTDSG-EKMTLSISVLLSLTVFLLVIVELIPSTSSAVPLIGKYMLFTMIFVISSIIVTVV   318
TOR_gamma    PAQAGGQKCTLSISVLLAQTIFLFLIAQKVPETSLNVPLIGKYLIFVMFVSLVIVTNCVI   319
             *  :.*  :* .:* **:**:  .**:::  :  :* **   :**:*:*:::::.      :    *.
                  __________________           ______________________
                         TM2                              TM3
                  C
Ce_ACR-2     IVNIYFRSALSHKMPTWVRKVFLEFLPHLLVMKRPERIPIFNGYFVEEYCASEIFDASLV   407
HU_alpha3    VLNVHYRTPTTHTMPSWVKTVFLNLLPRVMFMTRP-------------------------   359
TOR_alpha    VINTHHRSPSTHTMPQWVRKIFINTIPNVMFFS---------------------------   351
TOR_gamma    VLNVSLRTPNTHSLSEKIKHLFLEFLPKYLGMHLE-------------------------   354
             ::*   *:. :*.:.  :: :*:: :*. : :

Ce_ACR-2     MPSMTATMLPFLQVTTNLKAASSTSSGQSSEHHENCSKWKKRLSIRMSKRRAPRARLDDD   467
HU_alpha3    ----TSNEGNAQKPRPLYGAELSNLNCFSRAESKGCKEGYPCQDGMCGYCHHRRIKISNF   415
TOR_alpha    ----TMKRASKEK-----------------QENK---------------IFADDIDISDI   375
TOR_gamma    -PSEETPEKPQPRRRSSFGIMIKAEEYILKKPRSE----------LMFEEQKDRHGLKRV   403
                         :                    .                     :.

Ce_ACR-2     SEDIIDDTNGNHVDS--LQEKISKEMKTTVEAIAYIAEHMKREMSLKKMRDDWKYVAMVL   525
HU_alpha3    SANLTRSSSSESVDAVLSLSALSPEIKEAIQSVKYIAENMKAQNEAKEIQDDWKYVAMVI   475
TOR_alpha    SG---KQVTGEVIFQTPLIK--NPDVKSAIEGVKYIAEHMKSDEESSNAAEEWKYVAMVI   430
TOR_gamma    NKMTSDIDIGTTVDLYKDLANFAPEIKSCVEACNFIAKSTKEQNDSGSENENWVLIGKVI   463
             .        .  :                ::*  ::.  :**:  * :  .  .  ::*  :. *:
                                                                        _____
                      R                      X
Ce_ACR-2     DRLILLIFFGVTLGGTLGIICSAPHVFDFVDQEAIISKLNAKYLPSDMYS---   575
HU_alpha3    DRIFLWVFTLVCILGTAGLFLQP-----------LMAREDA------------   505
TOR_alpha    DHILLCVFMLICIIGTVSVFAGR-----------LIELSQEG-----------   461
TOR_gamma    DKACFWIALLLFSLGTLAIFLTG-----------HLNQVPEFPFPGDPRKYVP   505
             *:   : :    :    **  .::                :
             _______________
                   TM4
```
