# Supplementary figures and images for: A Neuronal Acetylcholine Receptor Regulates the Balance of Muscle Excitation and Inhibition in Caenorhabditis elegans
Source: PLoS Biol. 2009 Dec 22;7(12):e1000265. doi: 10.1371/journal.pbio.1000265 (PMC2787625; doi:10.1371/journal.pbio.1000265)

Supplementary Figure S2

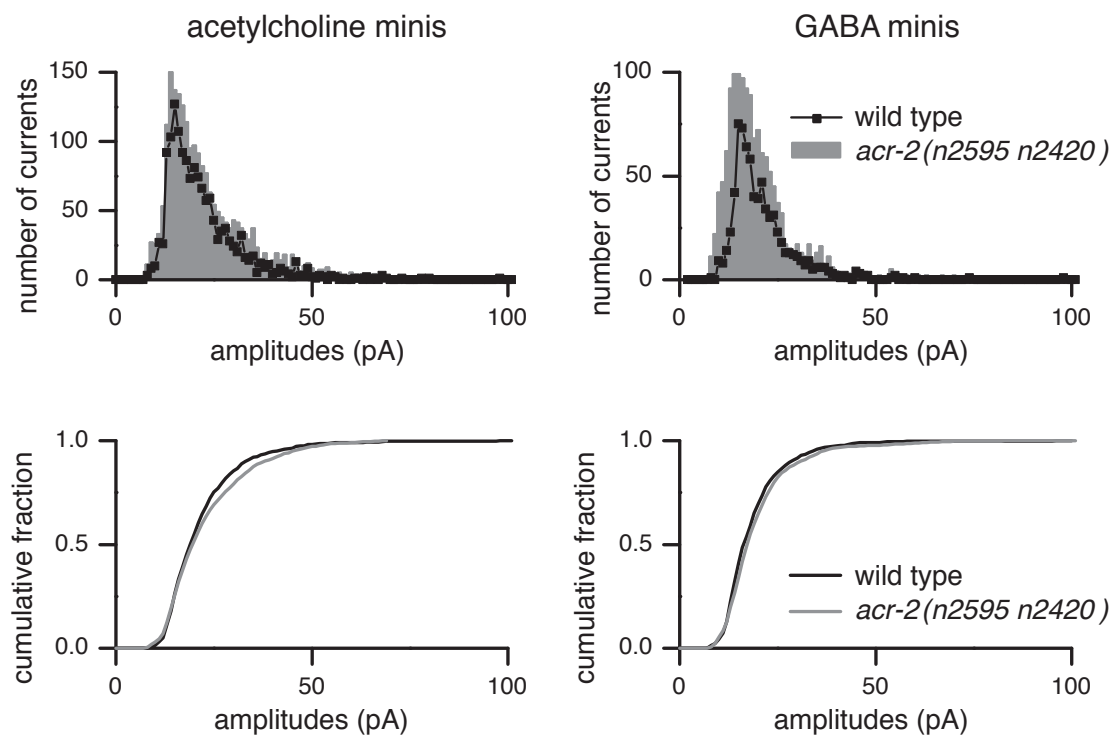

Supplement: Figure S2 — Mini amplitude is not altered in acr-2(n2595 n2420) . Amplitude histograms (top) and cumulative amplitude distribution (bottom) of acetylcholine (left) and GABA mini (right) from the wild type (n = 5) and acr-2(n2595 n2420) (n = 9) in 2 mM external CaCl2. (0.21 MB PDF) [file pbio.1000265.s003.pdf]

Supplementary Figure S3

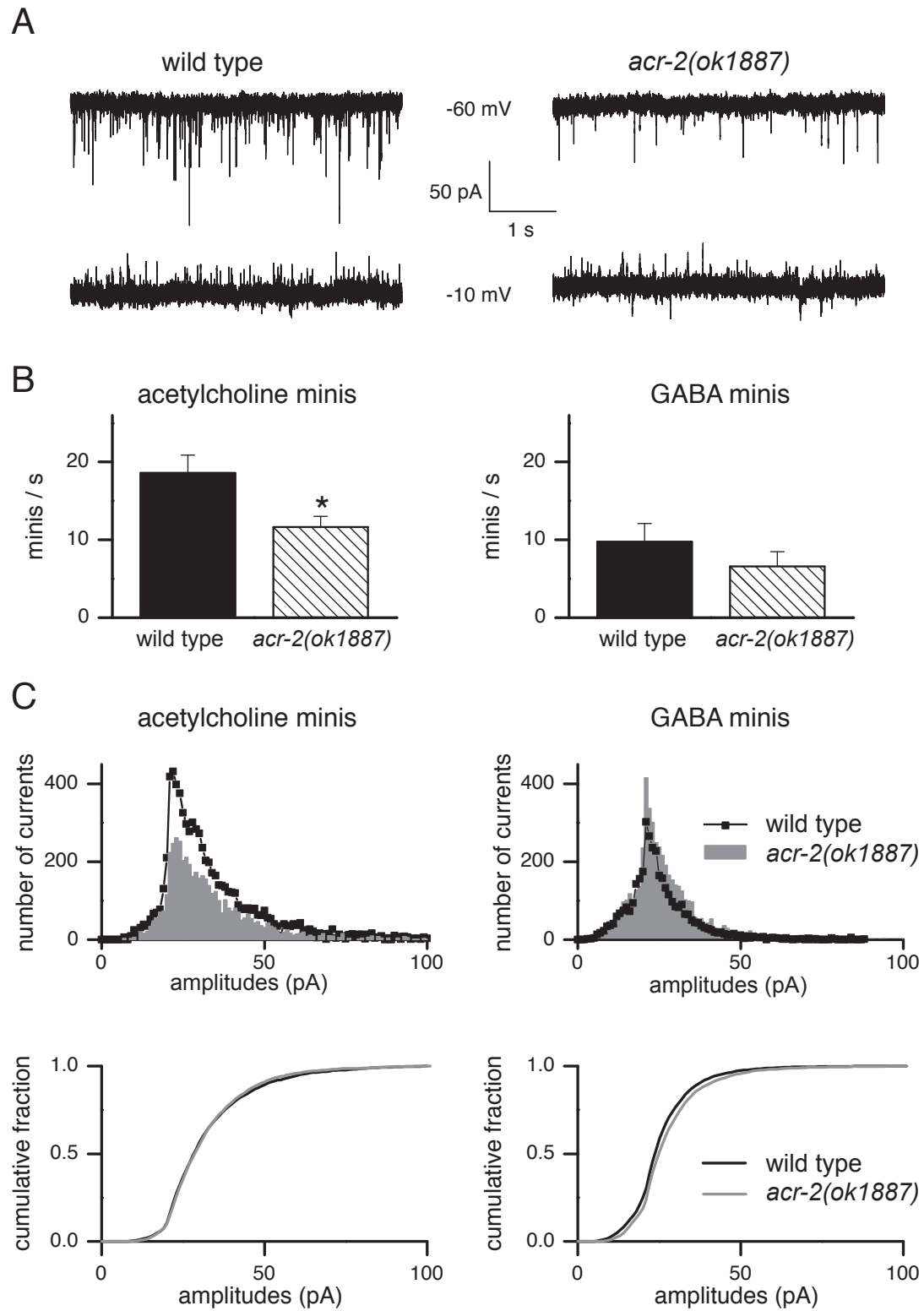

Supplement: Figure S3 — Acetylcholine neurotransmission is reduced in acr-2(ok1887) mutants. (A) Representative traces of minis recorded at two holding potentials, −60 and −10 mV, on body muscle cells from wild-type and acr-2(ok1887) worms in 2 mM external CaCl2. (B) Acetylcholine mini frequencies recorded in 2 mM external CaCl2 from the wild type (18.6 events/s±2.3 standard error of the mean [SEM], n = 8) and acr-2(ok1887) (11.6 events/s±1.4 SEM, n = 12) are significantly different (*p = 0.0144.). GABA mini frequencies recorded from the wild type (9.8 events/s±2.3 SEM, n = 8) and acr-2(ok1887) (6.6 events/s±1.9 SEM, n = 12) are not significantly different (p = 0.3003). Data were analyzed using a two-tailed unpaired t-test. (C) Amplitude histograms (top) and cumulative amplitude distribution (bottom) of acetylcholine (left) and GABA mini (right) from wild-type (n = 8) and acr-2(ok1887) (n = 12) worms in 2 mM external CaCl2. (0.98 MB PDF) [file pbio.1000265.s004.pdf]

Supplementary Figure S4

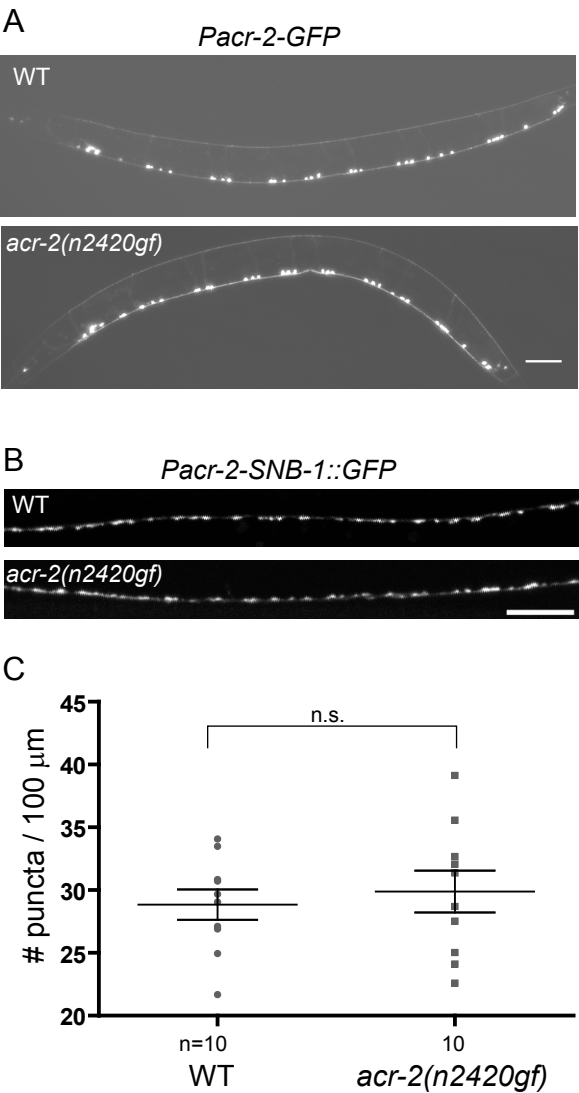

Supplement: Figure S4 — Cholinergic motor neuron morphology and synapses are not altered in acr-2(n2420gf) mutants. (A) Cholinergic A- and B-type motor neurons visualized with Pacr-2-GFP (juIs14) show normal position and morphology in acr-2(n2420gf) animals. Scale bar indicates 20 µm. (B) Pattern of DA and DB synapses visualized by Pacr-2-SNB-1::GFP (juIs20) is similar in acr-2(n2420gf) and wild-type animals. Scale bar indicates 10 µm. (C) Quantification of the SNB-1::GFP puncta number in a segment of the dorsal cord. N indicates the number of animals for each genotype. Statistics: unpaired Student t-test; error bars indicate the standard error of the mean; n.s., not significant. See Protocol S1 for image collection and analysis. (0.43 MB PDF) [file pbio.1000265.s005.pdf]

**A** *Punc-25:SNB-1::GFP*

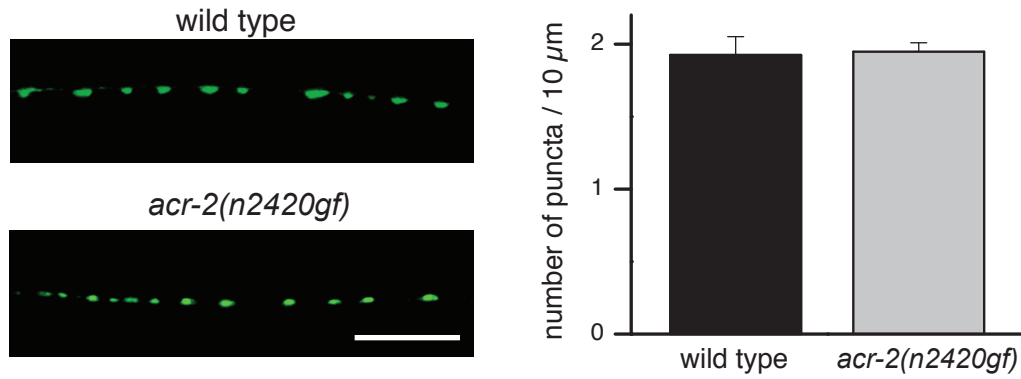

**B** postsynaptic GABA responses

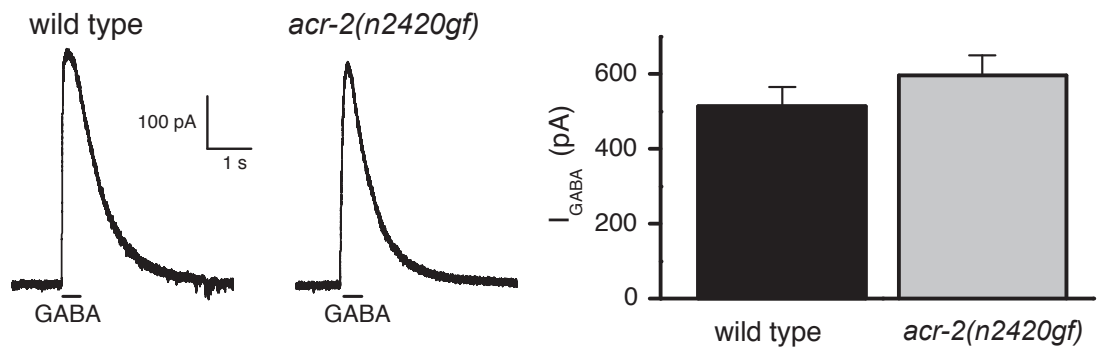

**C** acetylcholine minis

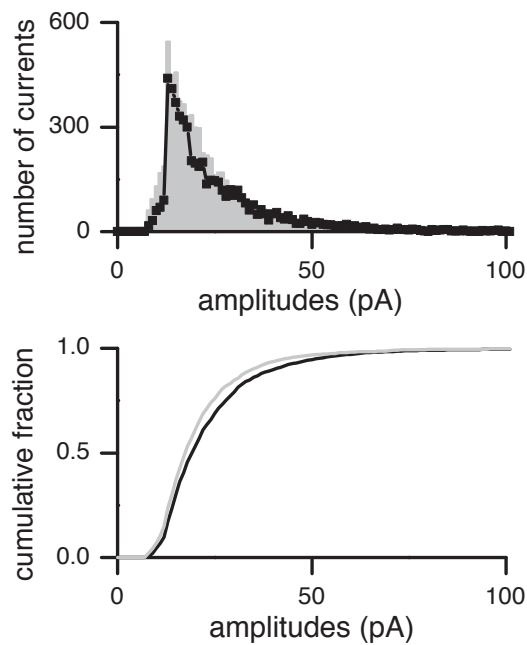

GABA minis

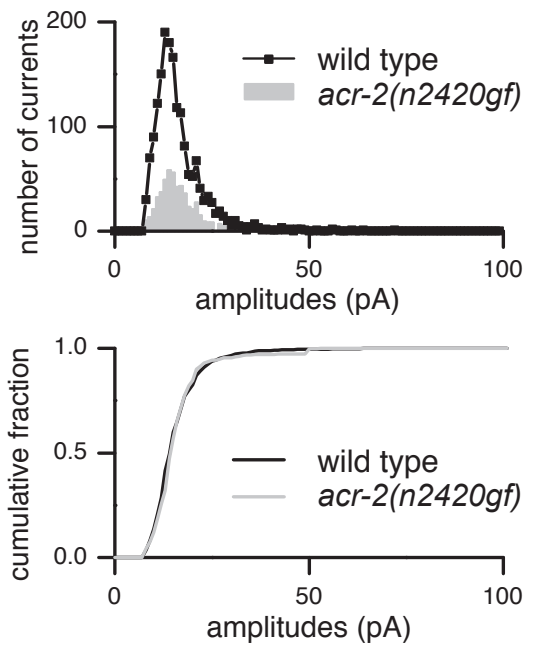

Supplement: Figure S5 — GABA receptors are not altered in acr-2(n2420gf) mutants. (A) Distribution of GABA synapses. Left panel shows the expression pattern of GFP-tagged synaptobrevin (Punc-25:GFP: synaptobrevin) in the dorsal cord of young adult wild-type and acr-2(n2420gf) animals, just posterior to the vulva. Quantification of the GFP puncta number is shown on the right. For wild type: 1.9 puncta/10 µm ±0.1 SEM, n = 8 and for acr-2(n2420gf): 1.9 puncta/10 µm ±0.1 SEM, n = 11. Scale bars indicate 10 µm. Data were analyzed using two-tailed unpaired t-tests. See Protocol S1 for image collection and analysis. (B) Amplitude histograms (top) and cumulative amplitude distribution (bottom) of acetylcholine (left) and GABA minis (right) from the wild type (n = 10) and acr-2(n2420gf) (n = 14) in 2 mM external CaCl2. (C) Representative traces and mean amplitude of currents evoked by 0.1 mM pressure-ejected GABA on muscle cells from the wild type (513.5 pA ±52.5 SEM, n = 6) and acr-2(n2420gf) (597.2 pA ±53.7 SEM, n = 14) in 2 mM external CaCl2. Mean amplitudes were compared using a two-tailed unpaired t-test. (0.52 MB PDF) [file pbio.1000265.s006.pdf]

Supplementary Figure S6

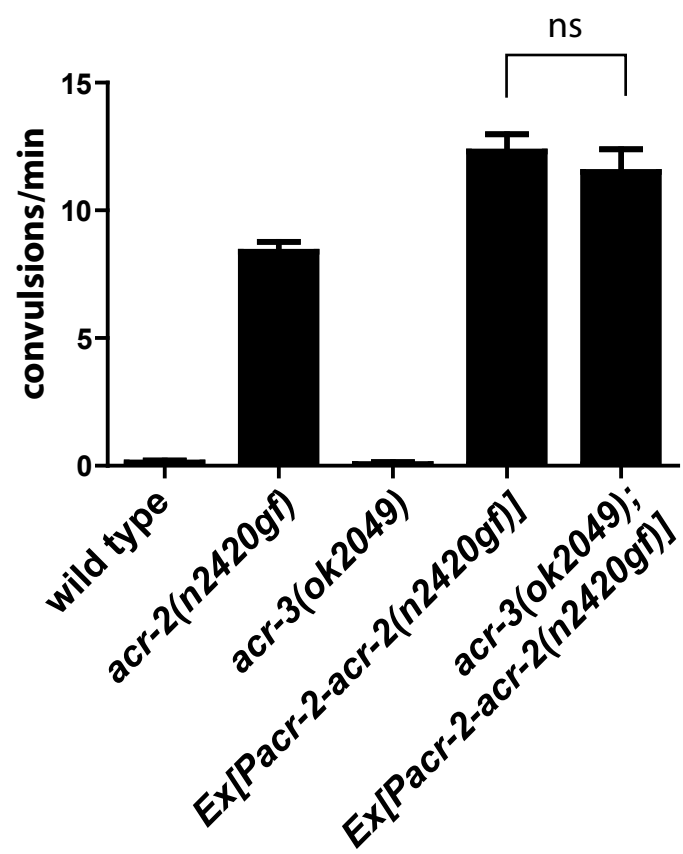

Supplement: Figure S6 — Loss-of-function acr-3 mutations do not suppress convulsions in transgenic animals expressing acr-2(n2420gf) . n = 10 animals per genotype. ns, not significant. See Protocol S1 for transgene construction. (0.35 MB PDF) [file pbio.1000265.s007.pdf]
