# Supplementary material for: A Neuronal Acetylcholine Receptor Regulates the Balance of Muscle Excitation and Inhibition in Caenorhabditis elegans
Source: PLoS Biol. 2009 Dec 22;7(12):e1000265. doi: 10.1371/journal.pbio.1000265 (PMC2787625; doi:10.1371/journal.pbio.1000265)
Supplement: Table S1 — Summary of suppressor mutations. (0.04 MB DOC) [file pbio.1000265.s008.doc]

Supplemental Table 1. Summary of suppressor mutations

| *Gene* | *allele* | Nucleotide change^1^/  Amino acid change | Gross behavior^2^ |
| --- | --- | --- | --- |
| *acr-2 X* | *n2420* | G925a  Val309Met | Unc, convulsion, hypersensitive to aldicarb and levimsole |
|  | *n2595*  *n2651** | G525a  Trp175TGA | Superficial WT, slight resistance to aldicarb, sensitive to levimosole |
|  | *n2581* | C529t  Pro177Ser | Superficial WT |
|  | *n2582*  *n2587** | g—a  3’ss, intron 5 | Superficial WT |
|  | *n2583* | C377t  Pro126Leu | Superficial WT |
|  | *n2584* | g—a  3’ss, intron 10 | Superficial WT |
|  | *n2585* | G631a  Glu211Lys | Weak Shrinker |
|  | *n2586*  *n2589* | C721t  Pro241Ser | Superficial WT |
|  | *n2588* | G617a  Trp206TGA | Superficial WT |
|  | *n2590* | C532t  Pro178Ser | Superficial WT |
|  | *n2591* | C199t  Leu67Phe | Superficial WT |
|  | *n2592* | C1669t  Gln557TAG | Superficial WT |
|  | *n2593* | g—a  5’ss, intron 11 | Superficial WT |
|  | *n2594* | C962t  Thr322Ile | Superficial WT |
|  | *n2598* | C614t  Ser205Leu | Superficial WT |
|  | *n2600* | C479t  Ser150Phe | Superficial WT |
|  | *n2601* | G682a  Asp228Asn | Superficial WT |
|  | *n2603* | T445g  Leu149Val | Superficial WT |
|  | *n2604* | C976t  Leu327Phe | Superficial WT |
|  | *n2628* | C1728t  Arg354Cys | Weak Shrinker |
|  | *n2631* | G698a  Gly233Glu | Superficial WT |
|  | *n2634* | C689t  Ser230Phe | Superficial WT |
|  | *n2635* | G1603a  Gly535Arg | Superficial WT |
|  | *n2636#* | A925g  Met309Val | Superficial WT |
|  | *n2602* | poss. rearrangement | Superficial WT |
|  | *n2596* | ND | Superficial WT |
|  | *n2597* | ND | Superficial WT |
|  | *n2599* | ND | Superficial WT |
| *unc-63 I* | *n2606* | A389t  Lys130Ile | Unc, resistant to levamisole |
|  | *n2607* | C475t  Pro159Ser | Unc, resistant to levamisole |
|  | *n2611^$^* | G361a  Gly121Arg | Superficial WT, weakly resistant to levamisole |
| *unc-38 I* | *n2608* | C333t  Pro111Leu | Unc, resistant to levamisole |
|  | *n2612* | g—t  5’ ss, intron 3 | Unc, resistant to levamisole |
|  | *n2615^$^* | C800t  Pro267Leu | Superficial WT |
|  | *n2619^$^* | G1430a  Gly477Glu | Superficial WT, slight resistance to levamisole |
| *unc-50 III* | *n2623* | C252t  Trp85TGA | Unc, resistant to levamisole |
|  | *n2624* | C782t  Ser261Leu | Superficial WT movement, slight resistance to levamisole |
| *unc-74 I* | *n2610* | ND | Unc, resistant to levamisole |
|  | *n2613* | ND | Unc, resistant to levamisole |
|  | *n2614* | ND | Unc, resistant to levamisole |
| *acr-12 X* | *n2616* | g—a,  3’ ss, intron 5 | Superficial WT  sensitive to levamisole |
|  | *n2617* | G684a,  Trp228TGA | Superficial WT  sensitive to levamisole |
|  | *n2621* | T769a,  Tyr256TAA | Superficial WT  sensitive to levamisole |
|  | *n2625* | G548a,  Trp183TAG | Superficial WT  sensitive to levamisole |
|  | *n2629* | g—a,  5’ ss, intron 10 | Superficial WT  sensitive to levamisole |
|  | *n2632* | g—a,  3’ ss, intron 10 | Superficial WT  sensitive to levamisole |
|  | *n2633* | G485a,  Cys162Tyr | Superficial WT  sensitive to levamisole |
|  | *n2650* | C905t,  Thr302Ile | Superficial WT  sensitive to levamisole |
|  | *n2652* | g—a,  3’ ss. Intron 5 | Superficial WT  sensitive to levamisole |

^1^ number of nucleotides uses the spliced sequences in wormbase for each gene

^2^  all animals contained *acr-2(n2420gf)*

* isolated as independent mutations

# this mutation was true revertant of *acr-2(n2420gf),* unlikely a contaminant of wild type

*^$^*  these mutants were weak alleles of Lev-Unc genes (T.S. and Y.J., unpublished data)

WT, wild type; Unc, uncoordinated
