# Supplementary material for: A Neuronal Acetylcholine Receptor Regulates the Balance of Muscle Excitation and Inhibition in Caenorhabditis elegans
Source: PLoS Biol. 2009 Dec 22;7(12):e1000265. doi: 10.1371/journal.pbio.1000265 (PMC2787625; doi:10.1371/journal.pbio.1000265)
Supplement: Table S2 — Strains and genotypes. (0.03 MB DOC) [file pbio.1000265.s009.doc]

Supplemental Table 2. Strains and genotypes

| Strain number | Genotype | Note on phenotype |
| --- | --- | --- |
| CZ707 | *lin-15(n765ts)X; juEx32[Punc-25-acr-2::gfp(pSC374); lin-15(+)]* | Superficial WT |
| CZ3393 | *juIs174[Pacr-2-acr-2::gfp(pSC349); pRF4]* | Roller |
| CZ4815 | *acr-2(n2420)X; juIs174* | Roller |
| CZ5259 | *acr-2(n2420)X; juEx32* | Shrinker |
| EG3867 | *acr-2(n2420)X; oxEx707[Pacr-2::acr-2(pSC175); lin-15(+)]* | Superficial WT |
| NC293 | *acr-5(ok180)III* | Superficial WT |
| CZ8840 | *acr-5(ok180)III; acr-2(n2420)X* | Shrinker |
| RB1195 | *acr-8(ok1240)X* | Superficial WT |
| CZ9228 | *acr-2(n2420)X acr-8(ok1240)X* | Shrinker |
| VC649 | *acr-9(ok933)X* | Superficial WT |
| CZ8937 | *acr-2(n2420)X acr-9(ok933)X* | Shrinker |
| VC188 | *acr-12(ok367)X* | Superficial WT |
| CZ8756 | *acr-2(n2420)X acr-12(ok367)X* | Superficial WT |
| RB918 | *acr-16(ok789)V* | Superficial WT |
| CZ8841 | *acr-16(ok789)V; acr-2(n2420)X* | Shrinker |
| CB211 | *lev-1(e211)IV* | Lev-resistant, Unc |
| CZ8919 | *lev-1(e211)IV; acr-2(n2420)X* | Lev-resistant, shrinker |
| VC1041 | *lev-8(ok1519)X* | Slight lev-resistant |
| CZ9006 | *acr-2(n2420)X lev-8(ok1519)X* | Shrinker |
| ZZ29 | *unc-29(x29)I* | Lev-resistant, Unc |
| CZ4796 | *unc-29(x29)I; acr-2(n2420)X* | Lev-resistant, shrinker |
| ZZ20 | *unc-38(x20)I* | Lev-resistant, Unc |
| MT7491 | *unc-38(e264)I; acr-2(n2420)X* | Lev-resistant, Unc |
| ZZ13 | *unc-63(x13)I* | Lev-resistant, Unc |
| MT7489 | *unc-63(e384)I; acr-2(n2420)X* | Lev-resistant, Unc |
| CB306 | *unc-50(e306)III* | Lev-resistant, Unc |
| CB883 | *unc-74(e883)I* | Lev-resistant, Unc |
| MF200 | *ric-3(hm9)IV* | Lev-resistant, Unc |
| CZ9103 | *ric-3(hm9)IV; acr-2(n2420X)* | Lev-resistant, Unc |
| ZZ17 | *lev-10(x17)I* | Lev-resistant, Unc |
| MT7493 | *lev-10(x17)I; acr-2(n2420)X* | Lev-resistant, shrinker |
| CZ631 | *juIs14[Pacr-2-GFP(pSC205); lin-15(+)]IV* |  |
| CZ8332 | *juIs223[Pttr-39-mCherry(pCZGY412)]* |  |
| CZ9210 | *acr-2(n2420)X acr-12(ok367)X; juEx1880[Pacr-12-acr-12*; Pttx-3-GFP]* |  |
| CZ9212 | *acr-2(n2420)X acr-12(ok367)X; juEx1882[Pacr-2-acr-12(pCZGY744); Pttx-3-GFP]* |  |
| CZ9214 | *acr-2(n2420)X acr-12(ok367)X; juEx1884[Punc-25-acr-12(pCZGY745); Pttx-3-GFP]* |  |
| CZ8901 | *unc-63(e384)I; acr-2(n2420)X; krEx164[Pmyo-3-unc-63#; Pmyo-3-GFP]* |  |
| CZ8902 | *unc-63(e384)I; acr-2(n2420)X; krEx181[Prab-3-unc-63#; Prab-3-GFP]* |  |
| CZ8903 | *unc-63(e384)I; acr-2(n2420)X; krEx201[Punc-63-unc-63#; Pmyo-3-GFP; Prab-3-GFP]* |  |
| CZ9216 | *unc-63(e384)I; acr-2(n2420)X; juEx1886[Pacr-2-unc-63(pCZGY746); Pttx-3-GFP]* |  |
| CZ9218 | *unc-63(e384)I; acr-2(n2420)X; juEx1888[Punc-25-unc-63(pCZGY747); Pttx-3-GFP]* |  |
| CZ9785 | *nuIs1[Pglr-1-GFP]; juEx2045[Pacr-2-mCherry]* |  |
| CZ9786 | *akIs3[Pnmr-1-GFP]V; juEx2045[Pacr-2-mCherry]* |  |
| CZ9610 | *juEx2033[Pacr-2-acr-2(n2420)*]* |  |
| CZ9755 | *acr-3(ok2049)X; juEx2033[Pacr-2-acr-2(n2420)*]* |  |
| CZ9690 | *acr-3(ok2049)X* |  |

*: generated as long-PCR fragment

#: described in Ruaud and Bessereau (2006)
